# Neuronal representation of visual working memory content in the primate primary visual cortex

**DOI:** 10.1126/sciadv.adk3953

**Published:** 2024-06-14

**Authors:** Jiancao Huang, Tian Wang, Weifeng Dai, Yang Li, Yi Yang, Yange Zhang, Yujie Wu, Tingting Zhou, Dajun Xing

**Affiliations:** ^1^ State Key Laboratory of Cognitive Neuroscience and Learning & IDG/McGovern Institute for Brain Research, Beijing Normal University, Beijing 100875, China.; ^2^ College of Life Sciences, Beijing Normal University, Beijing 100875, China.

## Abstract

The human ability to perceive vivid memories as if they “float” before our eyes, even in the absence of actual visual stimuli, captivates the imagination. To determine the neural substrates underlying visual memories, we investigated the neuronal representation of working memory content in the primary visual cortex of monkeys. Our study revealed that neurons exhibit unique responses to different memory contents, using firing patterns distinct from those observed during the perception of external visual stimuli. Moreover, this neuronal representation evolves with alterations in the recalled content and extends beyond the retinotopic areas typically reserved for processing external visual input. These discoveries shed light on the visual encoding of memories and indicate avenues for understanding the remarkable power of the mind’s eye.

## Teaser

Memory encoding in the primary visual cortex exhibits distinct patterns from sensory encoding and changes with recalled contents.

## INTRODUCTION

The exploration of subjective consciousness, a cornerstone of neuroscience, reveals the structure of our mental landscape. Manifestations of subjective consciousness are strikingly visual in nature (*1*–*4*), even in the absence of external visual stimuli. This visualization format is commonly attributed to the neural mechanisms of visual working memory (VWM) (*5*, *6*), our brain’s apparatus for the active maintenance and manipulation of informative images. Although VWM engages a widely distributed brain network (*7*–*11*), the fascinating nature of these near-tangible visualizations of memory and consciousness has steered the focus of neuroscience toward the visual cortex over the past decade (*12*–*18*).

Within this field, the role of the primary visual cortex (V1) has emerged as particularly controversial. While certain studies using functional magnetic resonance imaging (fMRI) suggest the existence of neural codes related to VWM content (*7*, *14*–*18*) in V1, others have proposed divergent views (*19*–*21*). Critically, the scarcity of previous electrophysiological evidence in V1 (*19*, *22*–*24*), especially concerning the core aspect of working memory—content rather than just spatial location (*12*, *13*)—emphasizes the need for further investigation. Unanswered questions remain regarding whether V1 neurons truly represent VWM content and, if so, how their activity contributes to such representation.

## RESULTS

To directly address these issues, we trained two monkeys to perform the delayed match-to-sample (DMTS) task (*9*, *25*) and recorded extracellular activity from V1 neurons during this task.

### Behavioral paradigm and V1 neuronal encoding of memory contents

**Fig. 1.**
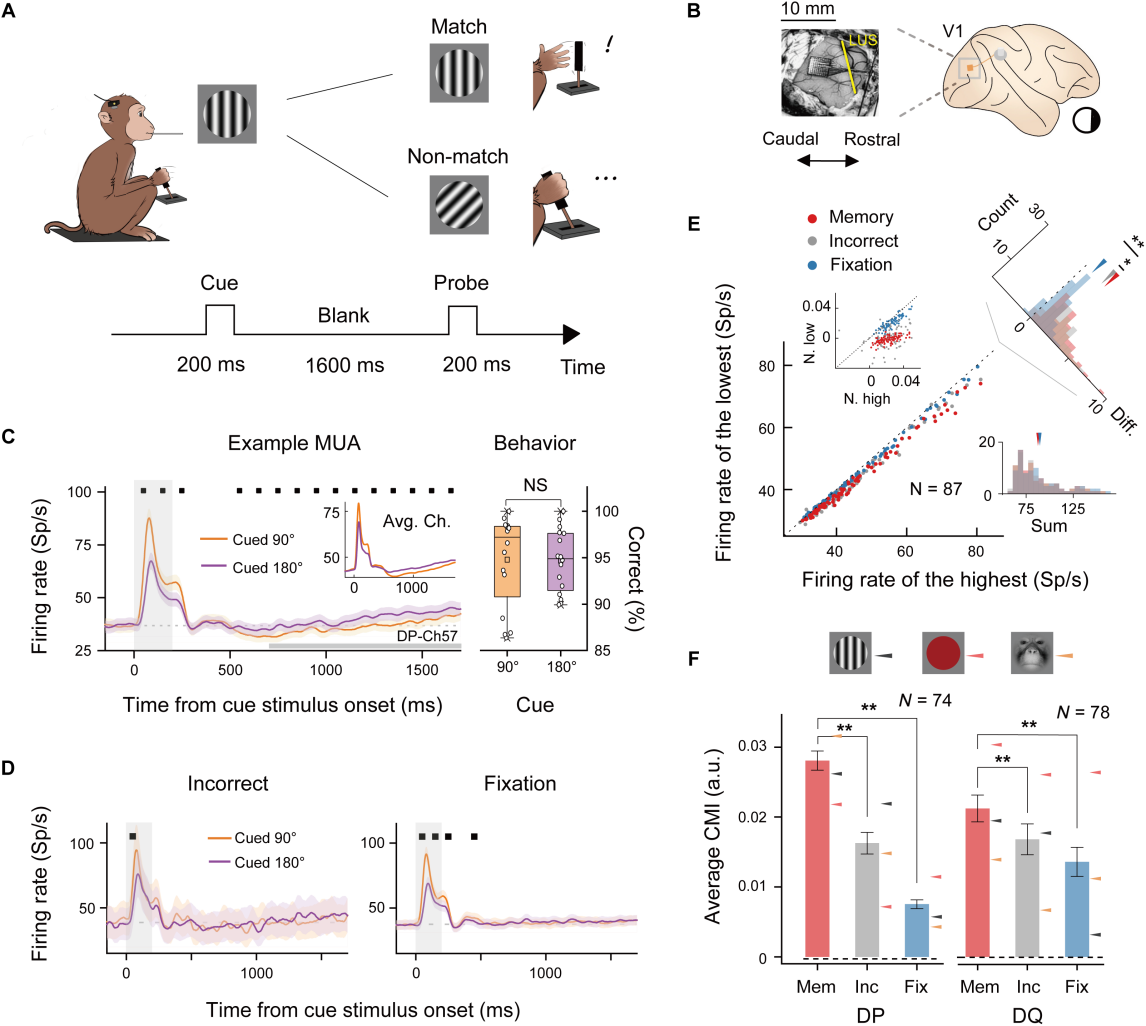
Behavioral task paradigm and neuronal encoding of memory contents. (**A**) Diagram of the orientation DMTS task. (**B**) Location of the electrodes in V1. LUS, lunate sulcus. (**C**) Left: Neuronal activity averaged across trials (*N* = 1810 for 90°, *N* = 1865 for 180°) from an example electrode. The horizontal gray bar defines the delay period. Right: Behavioral performance in the two stimulus conditions in the left panel. Avg. Ch. represents the averaged response across all channels. (**D**) Left: Neuronal activity from the same electrode as in (C) but for incorrect trials (*N* = 97 for 90°, *N* = 103 for 180°). Right: Neuronal activity in the fixation task (*N* = 1197 for 90°, *N* = 1276 for 180°). Black squares in (C) and (D) marked time periods with significant difference between the two conditions. (**E**) Comparison of averaged highest firing and lowest firing to VWM contents in the delay period for the three task conditions [in (C) and (D)] among all valid electrodes in monkey DP. The top right corner displays the difference between the highest and lowest firing, and the bottom right corner shows their sum. The inset graph shows the normalized responses (subtracting spontaneous activity and dividing by peak stimulus response). (**F**) CMI values for both monkeys in the three conditions during the delay period [as in (E)] in the orientation (black), color (pink), and face (yellow) tasks (DP: *N* = 74, DQ: *N* = 78 for electrodes valid in all three DMTS tasks). The dashed line represents the average CMI calculated from spontaneous activity. Mem, trials with correct answers; Inc, trials with incorrect answers; Fix, trials in the fixation task. All gray shaded regions indicate the stimulus period; all error bars represent ± SEM. **P < 0.01, *P < 0.05. Sp/s, spikes per second.

In the orientation DMTS task, the monkeys fixated on a small dot displayed in the center of the screen. In each trial, a stationary grating patch (i.e., the cue) was presented for 200 ms in one of four possible orientations (see Materials and Methods). After the cue, a 1600-ms blank screen was presented, ending with another 200-ms stimulus (i.e., the probe). Monkeys began each trial by depressing a lever on their chair and used the lever to indicate whether the probe stimulus shared the same orientation as the cue (Fig. 1A). Releasing the lever for matched probes or maintaining its depression for mismatched probes resulted in a sweet water reward. To control for memory demand, additional experimental blocks were conducted without any memory task or lever activation. In these blocks, although potentially engaging in the memory task mentally without active response requirements, monkeys were rewarded only for maintaining central fixation. We referred to this control experiment as the “fixation task.”

To capture a broader understanding of memory encoding, we expanded our experiments to include two other stimulus types: colors and face pictures (see Materials and Methods). Both monkeys demonstrated high accuracy in memorizing grating orientations in the “orientation DMTS” task, colors in the “color DMTS” task, and face pictures in the “face DMTS” task [DP: ~94% and DQ: ~87% versus 50%, all *P* < 0.01 (one-sample *t* test)] (fig. S1), indicating that they had been well trained.

We implanted a Utah array in each monkey’s V1 area (see Materials and Methods; Fig. 1B) and presented the stimuli onto the receptive field (RF) centers of the recorded neurons (fig. S2, A and D). This enabled simultaneous monitoring of neuronal activity in our experiments. Our analyses focused primarily on neuronal activity before probe stimulus onset.

Representative neuronal responses for two of the VWM content conditions in the orientation DMTS task at a selected electrode are shown in Fig. 1C. During the stimulus period (0 to 200 ms after cue onset), neurons displayed distinct firing patterns between the two content conditions (90° or 180° orientation). An off-response emerged following the cue offset, and activity gradually diminished. During the delay period, defined as 700 to 1700 ms after cue onset (the thick gray line in Fig. 1C), neurons also exhibited a significant difference in firing rate between the two content conditions (*N* = 1810 trials for 90°; *N* = 1865 trials for 180°; all marked positions *P* < 0.01) without any behavioral performance bias (*N* = 16 sessions, *P* = 0.94; right panel in Fig. 1C). The difference in response between these two content conditions during the delay period at the same electrode was less prominent in incorrect-response trials and in the fixation task (Fig. 1D).

This task modulation during the delay period was evident across most electrode sites (Fig. 1E; see fig. S3A for another monkey) [*N* = 87 electrodes, memory versus incorrect: *P* = 0.012; memory versus fixation task: *P* < 0.01 (paired *t* test)], meeting the criteria for brain regions that encode memory contents (*7*, *26*, *27*). Concurrently, no systematic increase in the V1 neuron firing rate was observed in the memory task during the delay period compared to the fixation task [*N* = 87 electrodes, both memory versus incorrect and memory versus fixation task: *P* > 0.05 (paired Wilcoxon signed-rank test)] (Fig. 1E; bottom right), suggesting that the difference in response to VWM contents likely arose from cue memory itself rather than from an increase in neuronal activity.

To exclude the possibility that the response during the delay period was induced by sensory adaptation from the response to the visual stimulus, which might have led to a negative response correlation between the two periods, we calculated the trial-by-trial response correlation between the stimulus period and delay period. We found that the response correlation was generally positive and close to zero (fig. S4), suggesting that the response in the delay period was unlikely to be affected by sensory adaptation.

We introduced a content modulation index (CMI; see Materials and Methods) to quantify the ability of V1 neurons to represent the content of VWM in both monkeys and across all memory stimulus types. Specifically, the CMI was the ratio of the difference to the sum of the firing rates for the highest and lowest content conditions in the delay period of memory trials at each electrode site. We found that the CMI values for correct-response trials were not only greater than those for spontaneous activity (0 to 150 ms before cue onset) but also greater than the CMI values for both incorrect-response trials and fixation task trials [time window: 700 to 1700 ms after cue onset; DP: *N* = 74, DQ: *N* = 78 for electrodes valid across all three DMTS tasks (orientation, color, face); all *P* < 0.01] (Fig. 1F; see fig. S3B for each experiment). These results revealed a memory-specific neural mechanism in V1 that underlies content-related distinctions during the delay epoch.

We next tested whether these content-related differences could resist interference from unrelated visual stimuli. In an independent experiment similar to the orientation DMTS task, we introduced a temporally random sequence of masking stimuli after the cue stimulus disappeared (fig. S5, A and B; see Materials and Methods for details). We observed that the representation of VWM content by V1 neurons could withstand interference from masking stimuli or immediately reappear after masking (fig. S5, C to E). This result also suggested that the VWM neural representation in V1 is not a by-product of the aftereffect of visual stimuli and may be supported by a top-down, interference-resistant mechanism.

To further validate our findings from multiunit activity (MUA), we examined the modulation of VWM content at the single-unit activity (SUA; see Materials and Methods). The CMI values calculated from SUAs (across all the DMTS tasks) also demonstrated that neuronal modulation was significantly greater than chance (permutation test; fig. S6) during the delay period. These results corroborate our MUA data, establishing that V1 neurons encode VWM content.

### A varying representation of VWM content revealed through neuronal population dynamics

Considering the inherent nature of coding sensory information by V1 (*28*), we next investigated whether V1 neurons use a coding strategy to represent VWM content similar to that used to represent stimulus content. To explore potential transformations in neuronal encoding during the DMTS task, we performed cross-validation analyses using decoding models (see Materials and Methods). The primary model used was the Poisson independent decoding (PID) model (*29*), with the linear support vector machine (SVM) model serving as an additional validation. Specifically, decoders trained on data from a specific time period during the DMTS task were used to decode memory content in other time periods (see Materials and Methods and fig. S7 for details). Training for the decoders began at time −200 ms (200 ms before the cue onset), with specific time steps (50 ms for the PID model and 200 ms for the SVM model) and a sliding window of 200 ms. This cross-validation analysis offers insights into the dynamics of VWM content coding by constructing separate classifiers using neuronal population activity within temporal sliding windows.

**Fig. 2.**
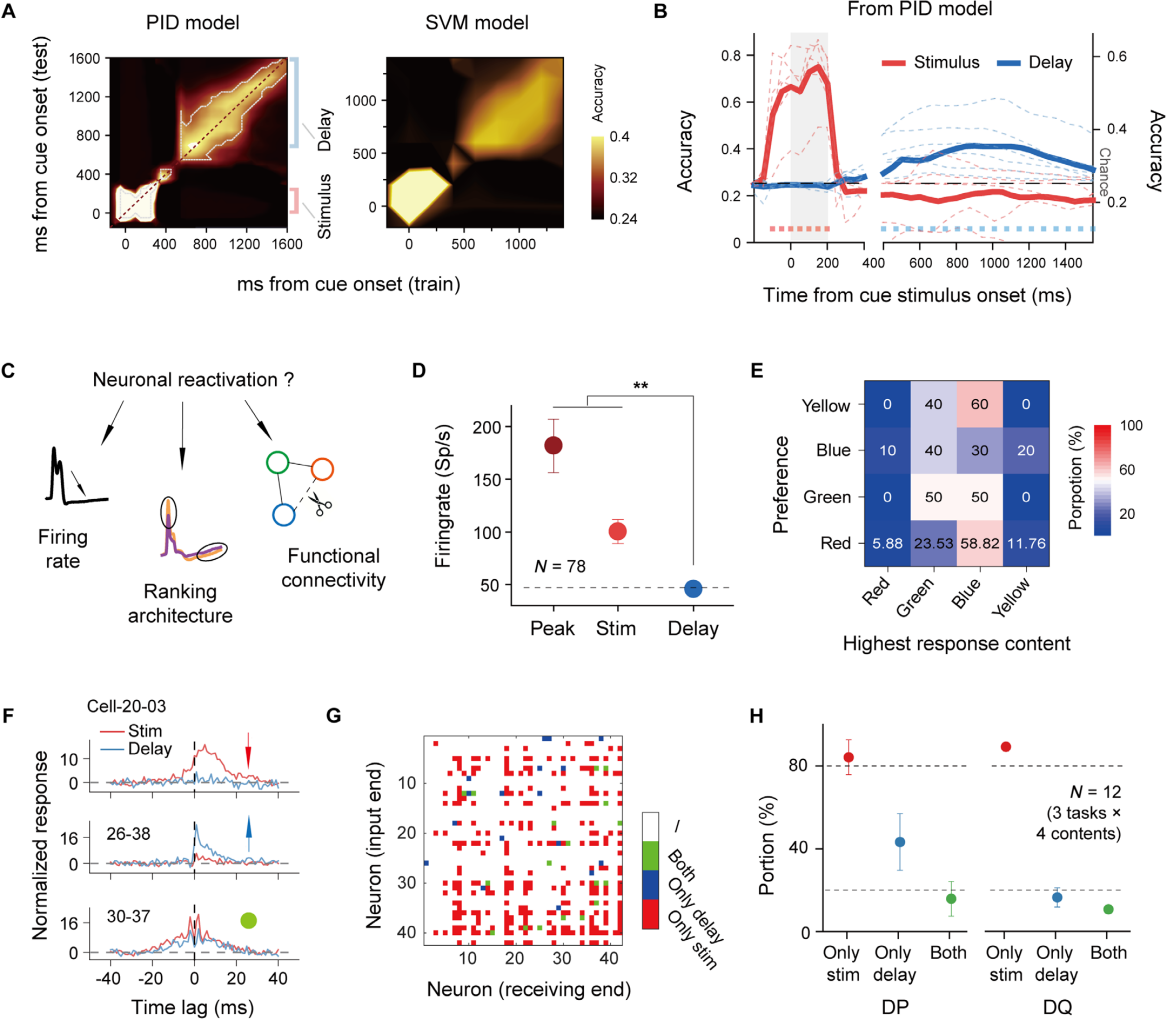
Varying representation of memory content in neuronal population. (**A**) Cross-temporal decoding of VWM content in DP’s orientation DMTS task. The left panel shows the results using the PID model, and the right panel shows the results using the SVM model. The chance level for decoding was 0.25. Significant decoding is indicated by a light gray dashed line (left panel, *P* < 0.05, permutation testing). (**B**) Averaged cross-sections from highlighted regions in the left panel of (A) for the stimulus period (red) and delay period (blue) in both monkeys [*N* = 6 experiments from two monkeys; *P* < 0.05 for all marked time windows (paired Wilcoxon signed-rank test)]. Different vertical scales are used for the stimulus (left) and delay (right) periods. (**C**) Illustration of three factors related to the reactivation of sensory encoding. (**D**) Response intensities of monkey DQ in the orientation DMTS task. The dashed line indicates the spontaneous level. (**E**) Proportion of neurons with a specific stimulus preference (row) that exhibited the highest response in the delay period (column) in DP’s color DMTS task. The percentage of neurons responding most strongly to a specific content during the delay period was shown as numbers and colors. (**F**) Examples of functional connectivity (FC) in the stimulus and delay periods for three FC categories. The vertical axis is scaled as multiples of the SD calculated from the efficacy during the ±20- to 40-ms time lag. (**G**) FC categories for neuron pairings in DQ’s orientation DMTS task under the 135° condition. (**H**) Proportions of the three FC categories displayed in (F) and (G), calculated relative to all significant FCs during the stimulus period. The 20% and 80% levels are indicated with dashed lines. All error bars represent ± SEM. **P < 0.01.

For clarity, we present a single PID cross-temporal generalization of the decoding results from the orientation DMTS task of monkey DP (Fig. 2A, left; see fig. S8 for all the experiments). The orientations of the grating were decodable not only during the stimulus period but also during the delay period, with on-diagonal decoding accuracy predominantly stronger than that expected by chance. However, VWM content in the delay period failed to achieve above-chance decoding when using the classifier constructed from neuronal activity in the stimulus period [pixels out of the dashed line, *P* > 0.05 (permutation test)], suggesting that neuronal encoding of visuals and memory in V1 has distinct formats. This result was also validated using the SVM decoder, which yielded very similar outcomes (see Fig. 2A, right).

For all the VWM experiments, we averaged horizontal cross-sections of the decoding accuracy matrix (of the PID model), specifically over 5 slices (time windows from 0 to 250 ms after cue onset) for stimulus periods (indicated by the red shading in Fig. 2A) and 18 slices (time windows from 700 to 1600 ms after cue onset) for delay periods (indicated by the blue shading in Fig. 2A) (see Fig. 2B). The decoding accuracy during the delay period remained above chance for within-slice decoding but not for cross-slice decoding [*N* = 6 experiments, all marked positions *P* < 0.05 (paired Wilcoxon signed-rank test)].

To further understand whether VWM representation is a reactivation of visual responses, we scrutinized three neuronal encoding factors: firing level, encoding ranking architecture, and neuronal functional connectivity (FC) (as illustrated in Fig. 2C). First, we compared neuronal firing rates during the stimulus period, including both peak and overall activity during the stimulus period, and during the delay period. We found that during the delay period, firing rates significantly decreased compared to those in the stimulus period, approximating levels of spontaneous activity (see Fig. 2D for DQ’s orientation DMTS results; see fig. S9 for all experiments). This finding suggested a lack of sustained elevated activation after the disappearance of visual stimuli. Second, we contrasted encoding rankings across these two periods, noting divergent highest response conditions for neurons during the delay period compared to the stimulus period (see Fig. 2E for DP’s color DMTS results; see fig. S10 for electrode-level data and fig. S11 for SUA). Furthermore, there was no significant correlation in the ranking of responses to VWM content conditions between the two periods (see fig. S12 and table S1). Third, we calculated the neuronal FC (*30*) for both the stimulus and delay periods. We performed cross-correlogram (CCG) analyses for all SUAs during these periods (see Materials and Methods). Figure 2F shows examples of three neuron pairs in DQ’s orientation DMTS: one pair exhibiting significant FC only during the stimulus period, another pair exhibiting significant FC only during the delay period, and a third pair exhibiting significant FC in both periods. The significance threshold (*31*) was defined as the maximum value within a 1- to 20-ms time lag exceeding seven SDs above the values during the ±20- to 40-ms time lag. We counted the numbers of these three types of FC pairs across all the memory content conditions (examples in DQ’s orientation DMTS are shown in Fig. 2G). Combining all DMTS task results from both monkeys, we found that approximately 80% of the neuron pairs with significant FC during the stimulus period were lost during the delay period. Conversely, during the delay period, approximately 20 to 30% of the different significant FC pairs emerged (Fig. 2H; *N* = 12 refers to the total number of conditions, combining four memory content conditions in each of the three DMTS tasks—orientation, color, and face—for both monkeys). This finding suggested that the FC between neuron pairs underwent notable changes between the stimulus and delay periods, suggesting dynamic changes in the local neural network at different phases of memory processing. These three observations strongly suggest that the neuronal representation of VWM contents is not reliant on the reactivation of neuronal populations associated with external visual processing within V1.

### Modulation of neuronal VWM representation according to content associations

In the aforementioned experimental context, neuronal encoding corresponded to the same external stimuli across both the stimulus and delay periods. In subsequent experiments, our aim was to determine whether V1 neuronal activity in the delay period is influenced by recalled content from long-term memory. This question extends beyond the basic function of neurons in merely preserving information, delving into the core attribute of working memory: the integration of external and internal information.

**Fig. 3.**
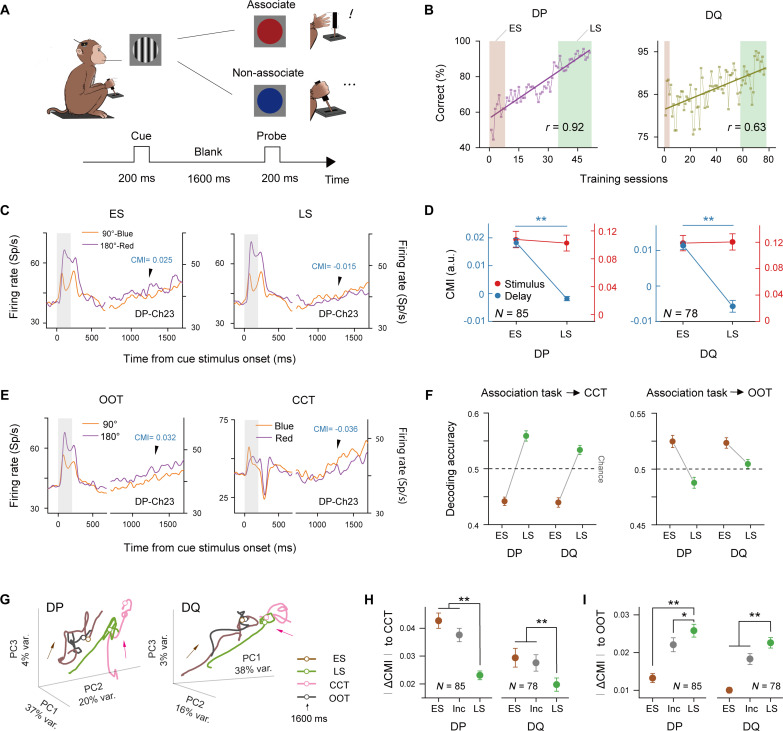
Neuronal activities reflect content associations. (**A**) Illustration of the association task. (**B**) Behavioral performance across training sessions. Shaded regions indicate early (ES) and late (LS) training stages. (**C**) Trial-averaged neuronal activity from an example electrode during the early (ES) and late (LS) training stages. (**D**) CMI values during the later delay period and stimulus period in the early and late stages (DP: *N* = 85 electrodes, DQ: *N* = 78 electrodes; for the later delay period, *P* < 0.01). (**E**) Neuronal activity from the same example electrode as (C) in the OOT and CCT. (**F**) Cross-validation results using SVM to decode memory contents in CCT and OOT, with decoders trained on population responses from ES to LS of the association task. Random iterations were performed 100 times. Error bars signify ± SEM, and the dashed line indicates chance level. (**G**) Projections of the CMI values onto the first three PCs (700 to 1700 ms after cue stimulus onset), with proportions of explained variance shown along each axis. Arrows indicate the direction of time in a trial. The hollow points represent the data at 1600 ms after cue stimulus onset. (**H**) Absolute CMI differences during the later delay period between the CCT and early/late association tasks. (**I**) Similar to (H) but for the OOT in comparison to the early/late stages of the association task. “ES” and “LS” refer to data from trials in the early and late stages of the association task, respectively, and “Inc” denotes trials where the monkeys answered incorrectly in the association task. All error bars represent ± SEM. *P < 0.05 and **P < 0.01.

To address this question, we trained the two monkeys to match an oriented grating stimulus to a specific color stimulus, which is referred to as the “association task.” After being cued with an oriented grating, the monkeys were required to judge whether a specific color stimulus was the probe (Fig. 3A). Each monkey had to learn two pairs of orientation and color stimuli (see Materials and Methods). We paired the condition (orientation) with the strongest population firing during the delay period of the orientation DMTS task with the condition (color) having the weakest population firing in the delay period of the color DMTS task, and vice versa (fig. S13). This antagonistic matching was designed to facilitate the observation of any change in potential neuronal representation during associative learning. We hypothesized that if the neuronal memory representation gradually shifts from orientation to color during associative learning, the neuronal population would show reduced or even reversed differences in response ranking between the two conditions during the delay period of the association task.

Before the association task, we evaluated the VWM neuronal representation of the specific colors used in the association task through a “color-color task” (CCT; see Materials and Methods). In this task, both the cue and the probe are colors. Notably, the CCT differs from the original color DMTS task, as the CCT’s visual stimuli only use two colors (red and blue) from the association task.

In the association task, the behavioral performance of both monkeys progressively improved as training sessions accumulated (Fig. 3B), enabling us to define the early and late stages (see Materials and Methods). This progression in behavior was paralleled by notable activity changes during the delay period. Specifically, at a representative electrode, the ranking of trial-averaged neuronal responses was consistent during the stimulus period across both the early and late stages but diverged during the later delay period (1200 to 1700 ms after cue onset) (Fig. 3C; see fig. S14A for response averaged across all electrodes). We quantified this difference in the neuronal population using CMI values of the stimulus period and the later delay period independently, calculated by consistently applying the highest and lowest neuronal response conditions determined in the early stage (see Materials and Methods). At the late stage of the association task, the CMI values of V1 neurons during the later delay period not only differed significantly from those in the early stage but also became negative, suggesting that the ranking of the neuronal population’s response to VWM content reversed, as hypothesized (DP: *N* = 85 electrodes, DQ: *N* = 78 electrodes; for both monkeys, *P* < 0.01). In contrast, the CMI values during the stimulus period remained unchanged (for both monkeys, *P* > 0.05) (Fig. 3D). This result indicates that content association affected neuronal memory maintenance activity but not sensory processing.

At the same electrode site illustrated in Fig. 3C, the ranking of paired-content conditions during the delay period of the late training stage was more similar to that found in the CCT (Fig. 3E, right). We used the data from the original orientation DMTS experiment (depicted in Fig. 1A) for additional comparison (Fig. 3E, left). In this context, we refer to the orientation DMTS experiment as the orientation-orientation task (OOT). To further investigate whether the changes in neuronal responses observed between the early and late stages were accompanied by shifts in population coding for memory contents (from orientation to color contents), we conducted cross-validation analyses (see Materials and Methods). Specifically, we used linear SVM decoders, which were trained during the later delay period of both the early and late stages of the association task, to decode color in the CCT and orientation in the OOT (see Fig. 3F). We found that during the early stage, VWM content representation was still biased toward orientation, but by the late stage, VWM content representation began to more closely resemble the representation of color memory in the CCT.

To visualize the change in response ranking of the neuronal population, a dimensionality reduction was conducted (*32*) on the trial-averaged CMI values of all the valid electrode sites (see Materials and Methods; Fig. 3G and fig. S14B). The similar response rankings observed between the association task and the CCT during the delay period, especially during the later delay period, in the late training stage hinted that the detected change might be attributed to the memory contents that the monkeys had learned to associate.

We quantified the difference in memory content modulation during the later delay period between the association task and the CCT by computing the absolute difference between the CMI values of each stage of the association task and the CCT for each valid electrode site (∣ΔCMI∣; see Materials and Methods). This approach helped estimate the stage of neuronal modulation of VWM content that was closer to the CCT based on CMI values. For instance, at the electrode shown in Fig. 3 (C and E), the CMI in the late stage (−0.015) was closer to that in the CCT (−0.036) than that in the early stage (0.025). According to the results across all electrodes, late-stage ∣ΔCMI∣ values were significantly lower than those of the early stage (for both monkeys, *P* < 0.01) and those when the monkeys answered incorrectly (Fig. 3H).

We computed the ∣ΔCMI∣ between the OOT and the association task as a baseline. For the OOT, the late-stage ∣ΔCMI∣ values were greater than those of the early stage (Fig. 3I; for both monkeys, *P* < 0.01). This trend contrasts with that observed for the CCT, which indicates that the monkeys’ growing task familiarity does not necessarily lead to a convergence in the neuronal representation toward an arbitrary memory content.

These findings suggest that in the late training stage, the neuronal representation of VWM content more closely approximated the original color-based VWM representation, highlighting an inclination toward the monkeys’ recalled content from long-term memory.

### Spatial scale for VWM content representations in V1

V1 is renowned for its ability to precisely perform retinotopic mapping of external input (*33*). However, the experience of retrieving a visual memory fundamentally differs from perceiving an external object, as these memory images may not remain stationary in our visual field (*34*–*36*). We wondered whether the representation of visual memory in V1 shares this spatial limitation with visual input or whether the representation was more expanded in space.

**Fig. 4.**
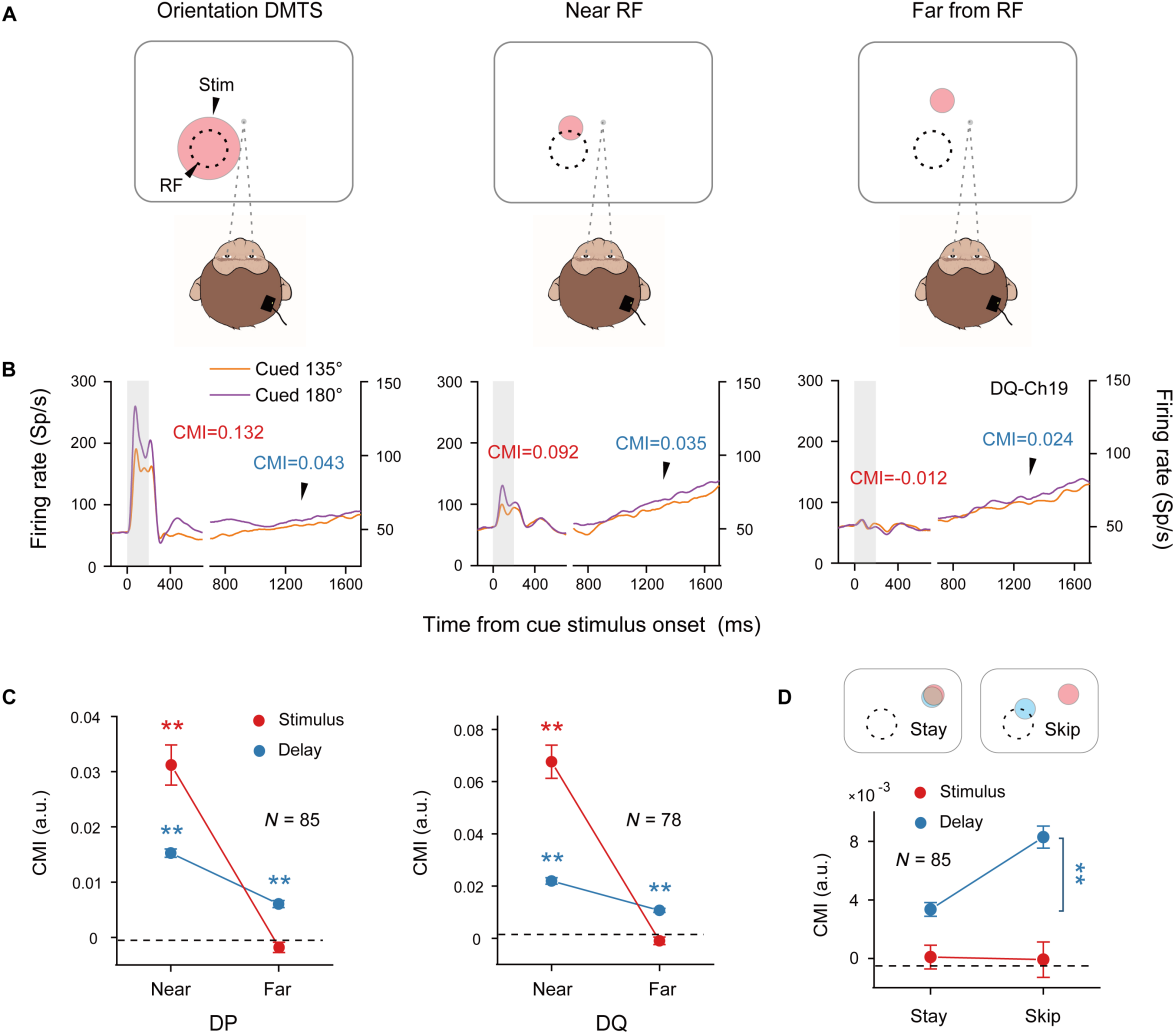
Spatial scale for VWM content representations in V1. (**A**) Stimulus positions relative to the spatial receptive fields (RFs) of neurons. (**B**) Time course of trial-averaged neuronal activity from an example electrode site corresponding to the three tasks in (A). Trial counts: “orientation DMTS”—1803 (135°), 2357 (180°); near RF—2192 (135°), 2492 (180°); far from RF—1959 (135°), 2127 (180°). (**C**) For both monkeys, CMI values were calculated from neuronal activity to quantify the differentiation of content representations in the near RF and far from RF conditions [DP: *N* = 85 electrodes, DQ: *N* = 78 electrodes, *P* < 0.01 (one-sample *t* test)]. (**D**) (Top) Stimulus location in the stay task and skip task. The light pink circle denotes the cue position, while the light blue circle indicates the probe position. Dashed circles represent the locations of the neurons’ RFs. (Bottom) During the delay period, the CMI values of neurons in the skip task were significantly greater than those in the stay task (*N* = 85 electrodes, *P* < 0.01). All error bars represent ± SEM, and **P < 0.01.

To address this question, we investigated the activity of neuronal populations at different locations relative to the visual stimulus, as detailed in Fig. 4A (the middle and right panels show the stimulus locations; for the detailed stimulus layout, see fig. S2). Tasks where a smaller stimulus (size at 1.2° in diameter) was positioned either near (approximately 1° between the stimulus center and the RF center) or far (approximately 2.5° between the stimulus center and the RF center) from the RFs of the neuronal population were labeled “near RF” or “far from RF”, respectively. These two tasks illustrate the range of near-to-far distances between the neuronal spatial RFs and the objective stimulus.

For comparison, we adapted the neuronal response from the original orientation DMTS as an example, where the stimulus covered the RF centers of the neuronal population (left panel in Fig. 4A). Example activities from the three aforementioned tasks, including the original orientation DMTS, are presented in Fig. 4B. In the original orientation DMTS task, neurons displayed a relatively high firing rate and clear differentiation of the two VWM contents during the stimulus period. However, in the far from RF task, the difference between contents vanished, accompanied by a low firing rate. The near RF task exhibited intermediate results. Remarkably, the ability to differentiate between VWM contents during the delay period was consistently maintained across all three tasks (the original orientation DMTS, the near RF task, and the far from RF task), despite the relatively low firing rates.

To quantify content differentiation at different distances between the RF centers and the stimulus center, we calculated the CMI values for the near RF and far from RF tasks. These two tasks, featuring the same stimulus size (1.2° in diameter), were analyzed for their CMI values during both the stimulus and the delay periods. In this calculation, we consistently applied the content conditions with the highest and lowest neuronal responses identified in the near RF task (see Materials and Methods). In the stimulus period, the CMI values for the far from RF task decreased to baseline [DP: *N* = 85 electrodes, DQ: *N* = 78 electrodes; for both monkeys, *P* > 0.05 in the far from RF condition (one-sample *t* test)], whereas they remained higher in the near RF task. This trend was less pronounced in the delay period [for both monkeys, *P* < 0.01 (one-sample *t* test)] (Fig. 4C). The trend of CMI values as a function of distance was further confirmed by the trend of decoding accuracy (by linear SVM decoding) as a function of distance (fig. S15A). Additionally, this neuronal representation of expanded VWM content does not originate from similar eye movements (fig. S15B).

On the basis of the above results indicating that the spatial extent of neuronal representation of VWM content was greater than that of visual stimuli, we further investigated whether subjective factors such as endogenous attention could alter this spatial extent. Specifically, we examined the potential spatial transfer of VWM content representation based on probe location. In an additional task, the cue was positioned at a contralateral position “far from the RF,” while the probe was situated “near the RF” (Fig. 4D and fig. S2, top; i.e., the “skip” task). Presumably, the monkey needed to focus on another predetermined location for the probe after observing the cue. In a comparative task, both the cue and probe stimuli consistently remained contralateral far from the RF (Fig. 4D and fig. S2, top; i.e., the “stay” task), eliminating the need for endogenous attention shifting. We calculated the CMI (see Materials and Methods) during the stimulus period and delay period separately for both the skip and stay tasks. During the delay period, the CMI values of neurons in the skip task were significantly greater than those in the stay task (*N* = 85 electrodes, *P* < 0.01) (Fig. 4D, bottom). No difference was detected in the CMI values between these two experiments during the stimulus period.

Collectively, these experimental findings suggest that VWM content representation encompasses different retinotopic extents of V1 neurons and that the topological assignment of VWM could be influenced by endogenous attention.

## DISCUSSION

Identifying neuronal representations of memory content in V1 may seem counterintuitive, given the challenges in consciously perceiving recalled images. One plausible explanation is rooted in the concept of perceptual thresholds (*37*), where the insufficiency of sensory cortex responses impedes the subjective reporting of awareness. In both previous research (*12*, *13*, *38*) and our findings, V1 neuronal firing during VWM delay was weaker than that triggered by external stimuli and approximated the level of spontaneous activity (fig. S9). This finding supports the growing consensus (*39*, *40*) that persistent elevated activity may not be essential for memory-specific responses in some brain regions. Other encoding formats, such as ramping-type firing in inhibited responses (*12*, *39*) or FC changes (Fig. 2H), might also play a role in encoding memory content. Another explanation for the challenge of recalling images with conscious perception could be that in awake primates, this perception necessitates V1 neuronal activity congruent with input encoding, which was not manifested in our results (Fig. 2E). Our data indicate a degree of preference in the V1 population for memory content (also aiding our antagonistic matching design in fig. S13B), distinct from their preference during sensory encoding (figs. S10 and S11). We speculate that the more uniform preferences in memory content across the V1 population may be influenced by feedback from higher brain regions related to animals’ long-term knowledge. This mismatch may help prevent memories from appearing as perceptible hallucinations, except when external vision is obscured, as occurs during dreaming (*41*) or psychedelic administration (*42*). Conversely, the relatively independent representation mode may contribute to the resilience of memory content representations to sensory interference, as demonstrated by the robustness of VWM representations even in the presence of intense masking (fig. S5) in our supplementary results. Our findings align more closely with the recent “rotational dynamic” hypotheses (*43*) and contrast with the narrowly defined “sensory recruitment” framework (*15*, *16*), which assumes a consistent neuronal firing pattern throughout the processing of VWM contents.

Notably, the memory content representation we observed has not been reported in earlier electrophysiological studies (*22*, *23*). Our study, in which significant VWM content modulation was detected at both the multiunit (Fig. 1C) and single-neuron levels (fig. S6), identifies two key factors for this discrepancy. First, the limited scope of neuron recording in earlier studies likely led to a focus on neurons that are more responsive to visual stimuli, even if they unmistakably identified a very small number of neurons exhibiting selectivity during the delay period (*44*). Future studies could benefit more from using advanced imaging techniques to explore a broader number/range of V1 neurons involved in memory encoding, which also reduce the impact of correlated sampling by Utah array as observed in our study (see fig. S13A). Second, the constraints of recording duration in early studies might have obscured memory-related neuronal modulation, particularly given the low firing rates of V1 neurons during memory maintenance. Our study performed months-long recordings at each site using a chronically implanted array, while previous studies generally conducted recordings at one site for just a few hours with acute single electrodes. Therefore, the challenge of detecting neuronal activity in a localized cortical area over a short duration should not be interpreted as negating the role of V1 in visual memory. In contrast, V1 neurons may engage in a more distributed manner to encode memories.

Making subjective associations better captures the essence of working memory than retaining an external image. In humans, subjective VWM content is typically processed with exceptional alacrity. However, in our study involving monkeys, the learning duration was prolonged in the long-term learning association task (*25*). Our findings in this experiment suggest that V1 neuronal activity evolves in response to changes in memory content (Fig. 3H). This provides evidence that the temporal dynamics within V1, even in the absence of sensory input, can be influenced by long-term memory recall. In the spatial dimension, we observed VWM content representations in V1 that extended toward the anticipated locations (Fig. 4D). This spatial tuning corresponds with recent VWM and internal representation theories in the visual cortex (*14*, *36*, *45*) and is postulated to confer spatial flexibility to working memory. Notably, the degree of flexibility may be dependent on the specific VWM task. When the memory target is the location itself, representations become relatively localized (*13*). This form of memory representation in V1 may stem from control exerted by other brain regions through feedback connections. We speculate that the hippocampus relays memory contents to the visual cortex, the frontal lobe modulates the intensity of memory representation, and the parietal region adjusts its position and shape. Together, they might slightly change the visual perception across the retinotopic field, as opposed to creating a fixed, detailed picture.

In our results, although the modulation produced by memory content during the delay period is relatively small in magnitude compared to the modulation of visual evoked responses, we believe that the encoding of memory content in V1 is crucial for the functioning of working memory. First, we observed that the encoding of memory content in V1 differs from the encoding of visual stimuli (Fig. 2), showing resistance to interference from external stimuli (fig. S5) and being influenced by associated content (Fig. 3). If the encoding of memory in V1 were not causally important, there would be no necessity for higher cortical areas to continuously modulate memory signals in V1, assuming the effect is top-down. Second, a more sparsely distributed cortical coding in V1 might enable memories to be effectively encoded without requiring substantial modulation in any single neuron. Still, our study does not provide a direct test of the causal relationship between neuronal encoding of VWM content in V1 and memory functions. Therefore, future research should explore how changes in V1 responses could affect visual memory, specifically by manipulating neuronal activity in V1 during periods of memory maintenance.

It is important to acknowledge the other limitations of our study. First, the variety of memory content in our experiments was limited, particularly in the association task. This limitation was partly due to the extensive time required to train the monkeys. Future studies with more diverse content conditions, if feasible, could better elucidate the encoding of VWM in V1. Second, in our supplementary analysis, we roughly estimated that the modulation range of memory content on neurons extends over a diameter exceeding 6° (fig. S16). However, our electrodes covered a relatively small area of the retinotopic map (see fig. S2), which limits accurate estimation of the exact spatial extent of memory representations. Although memory representations may have a flexible range, as shown in Fig. 4D, exploring the spatial structure of these representations and how they are influenced by endogenous attention is also crucial for future studies.

Our study reveals several additional intriguing questions. First, although we identified a neuronal encoding for VWM content that is separate from visual input, the permanence (or transience) of the memory content tuning remains uncertain. If this tuning is permanent, questions emerge about its development. If it is temporary, further exploration is needed to identify the brain region that is the source of these signals. Second, the dynamics of neuronal responses during VWM are not yet fully understood. How are the dynamics harnessed to facilitate the neuronal encoding of memory contents? What is the minimum scale of spatial-temporal data required to accurately reconstruct specific memory content (if augmented by artificial intelligence)? Considering these ongoing inquiries, the future of this field holds immense promise. We anticipate that our understanding of the potential depth of visual memory and the untapped possibilities within it will be greatly enhanced.

## MATERIALS AND METHODS

### Subjects

Two male macaque monkeys (*Macaca fascicularis* DP and *Macaca rhesus* DQ, aged 8 to 9 years at the beginning of the experiments and weighing 7 to 10 kg) participated in the experimental tasks. The monkeys were housed in a strictly temperature-controlled room that maintained a natural light-dark cycle. Licensed professionals with veterinary experience monitored the room’s temperature, humidity, and lighting conditions two to three times each day. The monkeys’ mental state, appetite, and metabolic waste were meticulously examined at the same frequency. The enclosure of each monkey was fully transparent, well ventilated, and provided ample space for exercise and movement. The monkeys could stand on tree branch–like perches and swing from horizontal bars at the top of their enclosures as desired.

To prevent injuries from potential fights, we limited the monkeys’ opportunities for extensive and intense physical contact in their housing setup. However, they could fully see each other and engage in all other social behaviors, such as gestures, vocalizations, and limited physical contact.

We ensured that the monkeys received sufficient solid food and fluid intake daily, monitoring their body weight to guarantee that any weight gain or loss remained within a reasonable range. In addition, we provided the monkeys with an abundance of fresh fruits, including but not limited to apples, bananas, cucumbers, carrots, and sweet potatoes.

On weekdays, the animals received sugar-diluted mineral water as a reward for correct responses during trials. We followed a carefully designed water intake regime to ensure that the monkeys received adequate hydration through the experimental setup. If any suspected physical or mental abnormalities occurred during the experimental days, we immediately suspended the experiments and initiated monitoring and potential subsequent treatment until the monkey fully recovered.

All our procedures and care for the monkeys adhered to the National Institutes of Health *Guide for the Care and Use of Laboratory Animals* and were approved by the Institutional Animal Care and Use Committee of Beijing Normal University [IACUC(BNU)-NKLCNL2022-08 and IACUC(BNU)-NKLCNL 2021-03].

### Task training

Fixation taskInitially, the monkeys were trained to fixate on a 0.3° circular dot, keeping their gaze within an invisible 2.3° window during all the experimental tasks. Trials where the monkeys’ eye movement deviated outside this window were immediately terminated, and the next trial commenced.

Color DMTS taskAfter completing fixation task training, the monkeys were trained to perform a color DMTS task. Initially, an 8°-diameter central color stimulus (i.e., the cue, typically in red) was displayed for 1000 ms, followed by a blank screen for 1000 ms. The monkeys were required to release the lever for a reward when a second stimulus (1000-ms duration) appeared on the screen. This phase familiarized the monkeys with a specific behavioral response to the second stimulus without necessarily involving mnemonic behavior. Subsequently, a second color (typically blue) was introduced as the probe stimulus. Monkeys learned to differentiate their lever responses based on probe color, maintaining the lever pull for an additional 400 ms following the blue probe presentation. Next, the blue color was incorporated into the cue with a 50% probability, requiring monkeys to judge whether the probe matched the cue color. After the monkeys fully mastered this task (achieving an ~90% correct rate), two additional colors (green and yellow) were sequentially introduced into the cue and probe sets, resulting in a 25% probability of each color being presented. Once the monkeys reached an ~90% accuracy rate, they proceeded to the next training phase.

Orientation DMTS task Upon completion of the color DMTS training, the monkeys began training with stationary oriented Gabor gratings as memory stimuli. Like in the color task, the initial training involved only two orientations (vertical and horizontal). Subsequently, 45° and 135° gratings were introduced as additional memory items.

Critical adjustment Following the color and orientation DMTS training, we reassessed the monkeys’ performance to ensure proficiency in both tasks. Subsequently, we gradually reduced the stimulus presentation time and increased the duration of the blank screens, ultimately reaching a 200-ms stimulus duration and a 1600-ms blank screen duration (as shown in Fig. 1A). We also trained the monkeys to respond after the probe stimulus disappeared, minimizing interference with the decision-making epoch during recording. Monkeys were required to release the lever within a 400-ms window after the probe stimulus offset if it matched the cue or to hold the lever for 1000 ms if it did not. Next, we reduced the stimulus size to 4° and ensured that the monkeys could correctly perform the DMTS task at appropriate peripheral locations while maintaining stable eye fixation.

Face DMTS task The training process for the face memory task was akin to that of the oriented grating memory task. The monkeys were able to master another DMTS task more rapidly, potentially due to behavioral generalization.

Masked DMTS task We introduced mask stimuli of the same size at the cue location during the delay period in the orientation DMTS task. Monkeys were required to disregard the mask’s appearance during the delay epoch. The mask could appear anytime between 200 ms after the cue offset and 200 ms before the probe onset. The masks consisted of five randomly oriented gratings or random colors, each lasting 20 ms with 80-ms intervals. In 10% of the trials, the mask did not appear. To maintain consistent global luminance throughout the trials, we equalized the physical luminance of all stimuli (cue, probe, mask, and background). To match the luminance of the colors, the average luminance of the oriented gratings in this task was lower than that in the original orientation DMTS task described above.

Orientation DMTS task at different locations After electrode implantation, we conducted this experiment and collected data before initiating association task training to prevent potential changes in neuronal memory representations. The monkeys rapidly adapted to this task, taking only approximately 3 days to learn it.

CCT Before the association task, we first administered the CCT to evaluate the neuronal representation of pure color in VWM. This task followed the same procedure as the color DMTS task. The key difference in the CCT was its exclusive use of color stimuli (red and blue), which were also used in the subsequent association task. Upon completing the CCT, we immediately proceeded with the association task the next day.

Association task Before the formal association experiment, we selected a pair of oriented gratings and colors based on the results of the abovementioned DMTS experiments. We measured the average population responses during the delay period and assigned the grating stimulus that elicited the strongest response as the cue and the color stimulus that elicited the weakest response as the probe, and vice versa. This antagonistic matching facilitates the observation of changes in potential neuronal representation during associative learning. Consequently, only two orientations were chosen as cues, and two colors were chosen as probes. We did not use any stepwise intermediate training. Instead, training sessions began with the monkeys familiar with the previous rule (responding only to stimuli in the same feature category). We directly replaced the probe stimuli with the corresponding color stimuli. The behavioral performance of both monkeys improved over time with accumulating training sessions (Fig. 3B). Monkey DQ learned rapidly, while monkey DP learned at a slower pace. Ultimately, both monkeys achieved an approximately 90% accuracy by the end of training.

### Stimulus details

The visual stimuli described below were displayed on a 22-inch cathode-ray tube (CRT) monitor (Dell, P1230; 1200 × 900 pixels; 100-Hz refresh rate) with a calibrated and linearized color lookup table (*46*).

The fixation point was a 0.3° circular dot, predominantly red except for tasks involving color stimuli, where it was white. Unless stated otherwise, the stimuli were presented as 4°-diameter circular patches, as shown in fig. S2 (A and D), and all the stimuli followed the temporal sequence depicted in Fig. 1A.

Orientation DMTS task Stationary sinusoidal gratings with four orientations (45°/90°/135°/180°) were used, featuring random phases (from eight phases equally spaced from 0° to 360°). The spatial frequency (SF) of the gratings was set at one cycle/degree for DP and two cycles/degree for DQ, with the aim of closely approximating the average optimal SF of each monkey’s V1 neurons. The mean luminance of all stimuli and the background was consistently maintained at 38 cd/m^2^.

Color DMTS task Patches with equal physical luminance featuring four colors (red/blue/green/yellow) were used (*46*). The mean luminance of all stimuli and the background was kept consistent at 8.5 cd/m^2^.

Face DMTS task Patches depicting four faces (two human faces and two monkey faces) were used in this task. The edges of the facial images were blurred to seamlessly transition into background. We ensured that no body parts beyond the face were visible in the images. The mean luminance of all stimuli and the background was held constant at 38 cd/m^2^.

Masked DMTS task The cue and probe stimuli used were identical to those used in the orientation DMTS task. We used two types of stimuli as masks (oriented gratings and color patches) with a 50/50 distribution. The grating masks consisted of sinusoidal gratings in eight orientations (equally spaced from 0° to 180°) with random phases (from eight phases equally spaced from 0° to 360°). The color patch masks featured 12 colors with equal physical luminance. The mask stimuli were presented as five rapid flashes, randomly occupying a 420-ms period within the period of the blank screen (300 to 1720 ms from cue stimulus onset). Each flash lasted 20 ms, followed by an 80-ms interval. The mask stimuli sizes were consistent with the cue and probe sizes, and the mean luminance of all the stimuli and the background remained constant at 8.5 cd/m^2^.

Orientation DMTS task at different locations The cue and probe stimuli in this task matched those in the orientation DMTS task, but they were positioned differently and had a reduced size (1.2° in radius). The stimulus locations and sizes are depicted in fig. S2, including the skip and stay tasks described in the main text. The mean luminance of all stimuli and the background was consistently maintained at 38 cd/m^2^.

CCT This task followed the same stimulus parameters and procedures as the color DMTS task, except the visual stimuli exclusively included the two colors (red/blue) used in the association task instead of the four colors used in the color DMTS task (red/blue/green/yellow).

Association task We used sinusoidal gratings of two distinct orientations (DP: 90°/180°; DQ: 135°/180°) with random phases (selected from eight equally spaced phases ranging from 0° to 360°) as the cue stimuli. Color patches in red and blue served as the probe stimuli. The mean luminance of all stimuli and the background remained constant at 8.5 cd/m^2^. The aforementioned four stimuli constituted two associative pairs: 90°-blue and 180°-red for monkey DP and 135°-blue and 180°-red for monkey DQ.

### Surgical procedures

During the initial surgery, the monkeys underwent implantation of a titanium head post under aseptic conditions and general anesthesia according to previously reported procedures. After a recovery period of at least 1 month and adequate rest, the monkeys were trained to perform the DMTS tasks described above. Subsequently, a second operation was conducted to implant 10 × 10 microelectrode arrays (Blackrock Microsystems) into V1. The arrays featured an interelectrode spacing of 400 μm and an electrode length of 1 mm. All procedures adhered to the National Institutes of Health *Guide for the Care and Use of Laboratory Animals* and were approved by the Institutional Animal Care and Use Committee of Beijing Normal University.

### Data acquisition and preprocessing

Electrical signals from the electrodes were amplified and digitized using a multichannel recording system (Blackrock Microsystems). MUA was detected by applying a voltage threshold with a signal-to-noise ratio (SNR) of 4.5 to the high pass–filtered (1000 Hz) signal. When measuring MUA spike rates, we set the MUA value as 1 for the time point at which the absolute value of the high-passed signal positively crossed (first exceeded) the threshold (i.e., transitions from below to above the threshold), and we set the MUA value as 0 for subsequent time points at which the absolute values of high-passed signal stay greater or less than the threshold. MUA represents neuronal spiking activities within a 100- to 150-μm radius of an electrode, and MUA population responses exhibit similarities to those obtained from pooled single units (*47*–*49*).

In light of previous findings indicating that V1 neuronal activity associated with working memory representation is relatively weak (*12*, *13*, *38*), we used a cautious strategy when devising our data collection plan. We mandated that for each specific memory task, a minimum of 500 correctly completed trials had to be collected for every memory content condition. This quantity greatly exceeded the data volume customarily acquired for observing activation due to external visual processing in V1, guaranteeing that we had sufficient resources for noise reduction and exhaustively exploring the possibilities related to our scientific inquiries.

Because of the inherent instability of the recording process, artifacts may have been present in the raw data. To ensure the reliability of the data and minimize the impact of the artifacts, we applied a stringent criterion by computing a firing rate distribution for all trials in each experiment. We excluded data from trials with a modified *z* score greater than 2.5 or less than −2.5 in this distribution, thereby maintaining high data quality (this exclusion criterion was not applied in the masking DMTS task due to a potentially larger variance in firing rate). The excluded data constituted less than 10% (5519/60,169 for monkey DP) and 12% (11,055/95,028 for monkey DQ) of the original data.

We determined the SNR for each electrode channel by dividing the peak height of the stimulus-evoked response by the SD of activity during the prestimulus period (−150 to 0 ms after cue stimulus onset). Electrode channels with an SNR less than five were excluded from the analysis. Furthermore, we manually excluded five problematic electrodes from monkey DQ due to substantial periodic mechanical noise.

### Data analysis

#### Receptive-field measurements

We used sparse noise (SPN) (*50*) to determine the spatial RF centers of the neurons. The SPN stimuli comprised dark and white squares (0.5° in width, contrast = 0.9) positioned at various locations across a square visual field approximately 5.5° wide. Each small square was displayed for 20 ms more than 50 times for both monkeys. We fitted the averaged responses within the stimulus field using a two-dimensional Gaussian function to estimate the center positions and sizes of the RF from each electrode site (MRF, 2σ of the Gaussian function). RF size ranged from 0.20° to 0.73° (median 0.30°), and eccentricity ranged from 1.65° to 3.10° in monkey DP and from 2.33° to 3.55° in monkey DQ. To determine a conclusive RF center for the array, we averaged the coordinates of the centers of all the well-fitted sites (goodness-of-fit for the Gaussian function > 0.6) (fig. S2; red square dots). This RF center informed the stimulus locations in our DMTS tasks. Notably, not all electrode sites included in the RF measurements were used in the formal experimental analysis, as some valid sites had less well-fitted RFs.

#### Content modulation index

We used a CMI to quantify the ability of neuronal activity to characterize distinct memory items. We designated the 0- to 200-ms interval following cue stimulus onset as the stimulus period and the 700- to 1700-ms interval as the delay period. The CMI was calculated independently for the stimulus period and the delay period. Additionally, as shown in Fig. 3 (H and I), we specifically calculated the CMI for the latter part of the delay period (1200 to 1700 ms after cue onset). For each electrode site and within both periods, we selected the conditions with the highest and lowest average firing rates (FRs) for CMI calculation as follows:

$${\text{CMI}}_{\text{site}}=\left({\text{FR}}_{\text{highest}}-{\text{FR}}_{\text{lowest}}\right)/\left({\text{FR}}_{\text{highest}}+{\text{FR}}_{\text{lowest}}\right)$$ (1)

In the above equation, FR_highest_ and FR_lowest_ represent the average firing rates of the conditions with the highest firing and lowest firing responses, respectively, within the specified time window of an experiment. When a result involves CMI values for both the stimulus period and the delay period, the CMI value for the stimulus period was calculated by designating the conditions with the highest firing and lowest firing responses during the stimulus period (0 to 200 ms after cue stimulus onset); then, the calculation is performed only using data within this stimulus period window. Similarly, the CMI for the delay period was calculated by designating the conditions with the highest firing and lowest firing responses during the delay period (700 to 1700 ms after cue stimulus onset); then, the calculation was performed only using data within this delay period window.

To maintain consistency and comparability of CMI values across experiments, we used a unified designation for calculations within the same task, using the conditions with the highest firing and lowest firing responses (h/l pair). This calculation method enables us to determine how much the content modulation in one experiment changes relative to the experiment used to obtain the h/l pair designation. For example, in the early stage of the association task, the average neuronal firing rate at an electrode site was highest for the 180°-red condition and lowest for the 90°-blue condition (as depicted in Fig. 3C). We designated these as a unified h/l pair for CMI calculations in the late association task and the CCT. Regardless of the specific stimulus condition exhibiting the highest firing rate in these two subsequent experiments, we used data from the 180°-red (red in color DMTS) as the “highest” (i.e., the minuend) and the 90°-blue (blue in color DMTS) as the “lowest” (i.e., the subtrahend) (Fig. 3, C and E).

Specifically, when comparing data between correct/incorrect DMTS trials and fixation tasks, we consistently used the h/l pair from the data from correct DMTS trials (Fig. 1F). When comparing data during/after the appearance of mask stimuli, we consistently used the h/l pair from the data before the first mask stimuli (0 to 100 ms before the first mask onset; fig. S5). When comparing data from different stages of the association task (orientation-color association test), the CCT and the OOT, we consistently used the h/l pair from the early stage of the association task (Fig. 3, D and G to I). When comparing the data at different stimulus locations, we consistently used the h/l pair from the near RF task (Fig. 4). Additionally, we tested the baseline CMI values of spontaneous activity (0 to 150 ms before cue stimulus onset) using the h/l pair from the data with correct memory decisions, and they were, on average, very close to 0 (see Figs. 1F and 4, C and D, and fig. S5; dashed lines).

Notably, this calculation, which employs two extreme conditions from one dataset to adapt to other datasets, requires two criteria to ensure the robustness of the results: inherent differences between conditions in the benchmark dataset and a substantial number of experimental repetitions. Our experiments were meticulously designed to satisfy both criteria. To validate the effectiveness of our CMI calculations, we implemented a methodological validation (see fig. S4C). We carried out a 20-fold cross-validation to ensure the random selection of a trial-number matched held-back dataset across all conditions of interest. In instances where the number of incorrect trials was less than 5% of the total, we adopted *k*-fold sampling based on the smallest ratio of incorrect to correct trials. The designations of the highest and lowest firing rates were extracted from a randomly chosen subset of correct trials in the DMTS task. We used these designations to compute the CMI values for each valid electrode in the held-back portion of correct memory trials, the trial-number matched subset of incorrect trials, and the trial-number matched subset of trials in the fixation task. This randomization process was repeated 200 times, and the CMI values from these iterations were averaged. The outcomes align with the findings in our manuscript. Notably, these results were tested on a relatively small dataset, constituting less than 5% of the total number of trials.

To quantify the similarity of neuronal memory representations between the association task and CCT and between the association task and OOT, we calculated the absolute value of the CMI difference (Fig. 3, H and I). For each electrode site, we obtained ∣ΔCMI∣ as follows

$$|\text{ΔCMI}|=|{\text{CMI}}_{\text{asso}}-{\text{CMI}}_{\text{CCT}}|$$ (2)

where CMI_asso_ represents the CMI in the association task (including the early stage and the late stage), while CMI_CCT_ denotes the CMI in the CCT. The calculation of the absolute value of the CMI difference between the association task and the OOT was conducted in the same manner.

#### Spike sorting

For each DMTS experiment conducted on two monkeys, spike sorting was performed with KiloSort software (*51*). The quality of each sorted unit was assessed by the SNR of the spike waveform. This SNR is defined as the ratio of the peak-to-peak amplitude of the mean waveform to twice the SD of the noise (*52*). Units with a spike waveform SNR greater than 1.5 were selected and classified as single units (SUAs).

#### Cross-temporal decoding

First, we computed the average firing rates of 20 adjacent trials with identical memory content, grouping them as a batch. Next, we applied a sliding window with a width of 200 ms and a step size of 50 ms along the temporal axis to average the firing rates within each batch. These two steps yielded a more robust dataset.

The neuronal population response matrix was constructed as *X* = (*X*_1_, …, *X_N_*)*^T^*, where *Xi* is an *m* (*m* being the total number of batches) by 1 vector of the averaged MUA response from electrode site *i* in a given time bin. *N* represents the number of electrode sites included. We randomly selected 60 batches (i.e., allocating 15 batches from each labeled content condition) from this matrix as the training set and used the remaining batches as the test set.

We used the PID model (*29*, *53*) to compute the overall log-likelihood function for estimating the content label

$$\text{log}L\left(\theta \right)=\sum_{i=1}^{N}n_{i}\text{log}f_{i}\left(\theta \right)-\sum_{i=1}^{N}f_{i}\left(\theta \right)$$ (3)

where θ denotes the stimulus and neurons tuned to stimulus θ*_i_* fire *n_i_* spikes in a given time window. *f_i_*(θ) represents the condition tuning of neurons in a given electrode and serves as the weight. The overall log likelihood of a stimulus at any θ can be calculated as a weighted sum of the neuronal response. In our decoding process, the log-likelihood function used the neuronal activity *n_i_* in the test data, weighted by the logarithm of the tuning function log*f_i_*(θ) derived from the training data (fig. S7A). We pooled the log-likelihood function log*f_i_*(θ) from all valid electrodes. Following procedures established in a previous study (*53*), we subtracted the bias term, represented by the averaged tuning function *f_i_*(θ) of all the electrode sites (the second term in Eq. 3, which was also calculated from the training data), from this sum.

The point at the peak of log*L*(θ) was selected as the estimated stimulus (fig. S7A). To evaluate the decoding accuracy, we determined the proportion of the test set that was correctly classified. In Fig. 2A (left), significance was assessed using a one-tailed permutation test to compare against the shuffled-label chance level. A decoding accuracy below chance implies that the neuronal population in the given time window does not represent memory content using a consistent encoding pattern between the test data and the training data, while an accuracy of 1 indicates ideal and consistent memory content representation.

In each experiment, we calculated the weight log*f_i_*(θ) for every time bin in the training set and applied them to all time bins in the test set for decoding (fig. S7B). This decoding process was repeated 1000 times, generating an averaged two-dimensional matrix of decoding accuracies [as shown in Fig. 2A (left) and fig. S8, color filling based on contour levels without additional smoothing].

#### CCG analysis

To estimate FC among neurons, we computed the cross-correlation between spike trains of all pairs of single units obtained through spike sorting. CCGs (*30*, *31*) were calculated separately for each memory content condition in the DMTS tasks of both monkeys. Consequently, for each monkey, there were 12 (4 content conditions in each DMTS task) sets of CCGs, each for two periods: stimulus (0 to 200 ms after cue onset) and delay (700 to 1700 ms after cue onset). We normalized the cross-correlation for each neuron pair by the geometric mean of their firing rates during the stimulus and delay periods. This normalization was performed to mitigate firing rate effects.

The computation of the CCG for a pair of neurons (*j, k*) in a specific memory content condition *c* is as follows

$$\text{CCG}{\left(\tau \right)}_{j,k,c}=\frac{\sum_{i=1}^{M}\sum_{\tau +1}^{N}r_{j}^{i}\left(t-\tau \right)\times r_{k}^{i}\left(t\right)}{\sqrt{\sum_{i=1}^{M}\sum_{\tau +1}^{N}r_{j}^{i}\left(t-\tau \right)\times \sum_{i=1}^{M}\sum_{\tau +1}^{N}r_{k}^{i}\left(t\right)}}$$ (4)

where *M* represents the number of trials for a specific content condition *c*, *N* denotes the number of time bins within a trial, τ is the time lag between the spike trains of the paired neurons, and $r_{j}^{i}\left(t\right)$ is the firing of neuron *j* in time bin *t* of trial *i*.

To correct for correlations attributable to stimulus locking or slow fluctuations in population response, we subtracted an average of jittered CCGs from the original CCG. These jittered CCGs reflect the expected values computed from all possible spike train jitters within a specified jitter window, preserving both the peristimulus time histogram (PSTH) of the original spike train across trials and the spike count within each trial’s jitter window. This jitter correction removes correlations between PSTHs (due to stimulus locking) and correlations on timescales longer than the jitter window (such as slow population correlations). We selected a 25-ms jitter window for this correction, in line with previous studies (*31*). Additionally, we conducted three rounds of jittered CCG calculations and averaged these three jittered CCGs to obtain the final jittered CCG used for correction. This approach helped to avoid introducing noise from jittered CCGs.

Finally, we determined a CCG to be significant if the maximum value of the jitter-corrected CCG within a 1- to 20-ms time lag exceeded seven SDs above the values during the ±20- to 40-ms time lag. This method allowed us to robustly identify significant neuronal connectivity beyond chance-level fluctuations.

#### Defining early and late stages in the association task

We defined the “early stage” as the sessions covering the initial 1000 correct trials during the association task. We defined the “late stage” as the sessions starting from the end and moving backward to the first instance where the average accuracy across the examined sessions fell below 90%.

#### PCA visualization

To visualize CMI differences between the early and late stages of the association task and the CCT (Fig. 3G), we used dimensionality reduction, as performed in previous studies (*32*). We first pooled the CMI values calculated from all valid electrodes in the association task (combining the early- and late-stage data). We then averaged these pooled CMI values and calculated their principal components (PCs). This dimensionality reduction transformed a matrix of electrode number × time into a matrix of PC number × time. Consequently, we were able to establish a transformation relationship between the electrode CMI values and the first three PCs. This relationship enables us to project any set of CMI values (from the corresponding electrodes) into a unified three-dimensional space. Finally, we projected the averaged activity from both the early and late stages of the association task, as well as the CCT, onto the first three PCs. Given that the PC space might differ in the CCT, we also computed the PCs using the CMI of the CCT and performed the same projection process (fig. S14B). To aid visualization, the resulting trajectories were interpolated and smoothed using a 100-ms boxcar filter. Statistical analyses of the Δ^2^ CMI (Fig. 3, H and I) were conducted using original data from all the valid electrode sites rather than from the visualized PCs, preventing any information loss due to principal components analysis (PCA) or smoothing.

#### Cross-task generalization validation

We used a linear SVM model to classify the content conditions. The model was trained using MATLAB’s fitcecoc function, with parameters optimized automatically via the bayesopt function. In preparing the training and testing sets, we randomly selected 300 trials from each content condition for the training dataset. We then averaged the neuronal responses during the specific time window under analysis. For the testing phase, we randomly chose 300 trials from each condition in the test dataset. This process of random iteration was repeated 100 times. The weights of the trained model were applied to this subset of the test dataset. Decoding accuracy was determined by calculating the proportion of correctly estimated content labels by the decoder compared to the actual content labels of the test set.

#### Eye position

We used an infrared tracking system (ISCAN) to monitor the left eye movements of the monkeys during the tasks. The system featured a sampling rate of 120 Hz. The average eye position eccentricity during the delay period in all DMTS tasks did not exceed 0.1° for either monkey.

#### Statistical procedures

All the statistical tests used two-tailed paired or unpaired *t* tests, except where noted below. We used one-sample *t* tests to compare the monkeys’ performance against chance levels in all the memory experiments, ensuring that both monkeys performed above chance in each task. To determine whether firing rates in memory tasks were greater than those in tasks with lower memory demands, we applied the one-tailed paired Wilcoxon signed-rank test. To assess the statistical significance of the differences in decoding accuracy, we conducted one-tailed permutation tests and paired Wilcoxon signed-rank tests for comparisons to chance. To evaluate the degree of similarity between neuron encoding rankings during the stimulus and delay epochs, we used Spearman’s rank correlation analysis. All error bars and dispersion measures are presented as means ± SEMs.
