# Supplementary material for: Neuronal representation of visual working memory content in the primate primary visual cortex
Source: Sci Adv. 2024 Jun 14;10(24):eadk3953. doi: 10.1126/sciadv.adk3953 (PMC11177929; doi:10.1126/sciadv.adk3953)
Supplement: Supplementary file 1 — Figs. S1 to S16 Table S1 [file sciadv.adk3953_sm.pdf]

Science Advances
AAAS

Supplementary Materials for

Neuronal representation of visual working memory content in the primate primary visual cortex

Jiancao Huang *et al.*

Corresponding author: Dajun Xing, dajun_xing@bnu.edu.cn

Sci. Adv. **10**, eadk3953 (2024)
DOI: 10.1126/sciadv.adk3953

This PDF file includes:

Figs. S1 to S16
Table S1

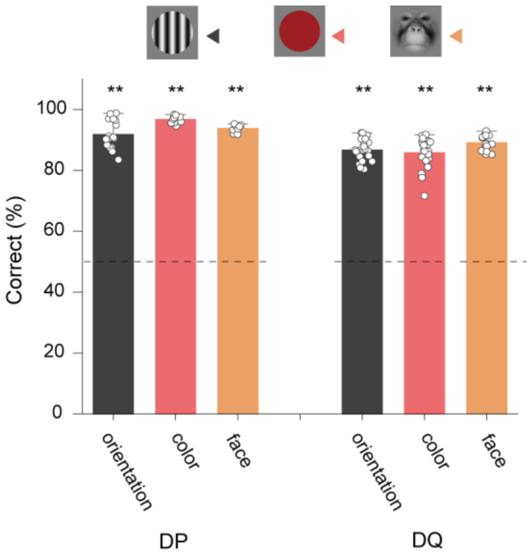

fig. S1. Behavioral performance of both monkeys on DMTS tasks. The bar colors represent the materials used in the DMTS tasks (black: orientation DMTS; red: color DMTS; yellow: face DMTS). Both monkeys exhibited strong performance in all DMTS tasks [DP: ~94%, DQ: ~87%; all $P < 0.01$ (one-sample t test vs. 50%)].

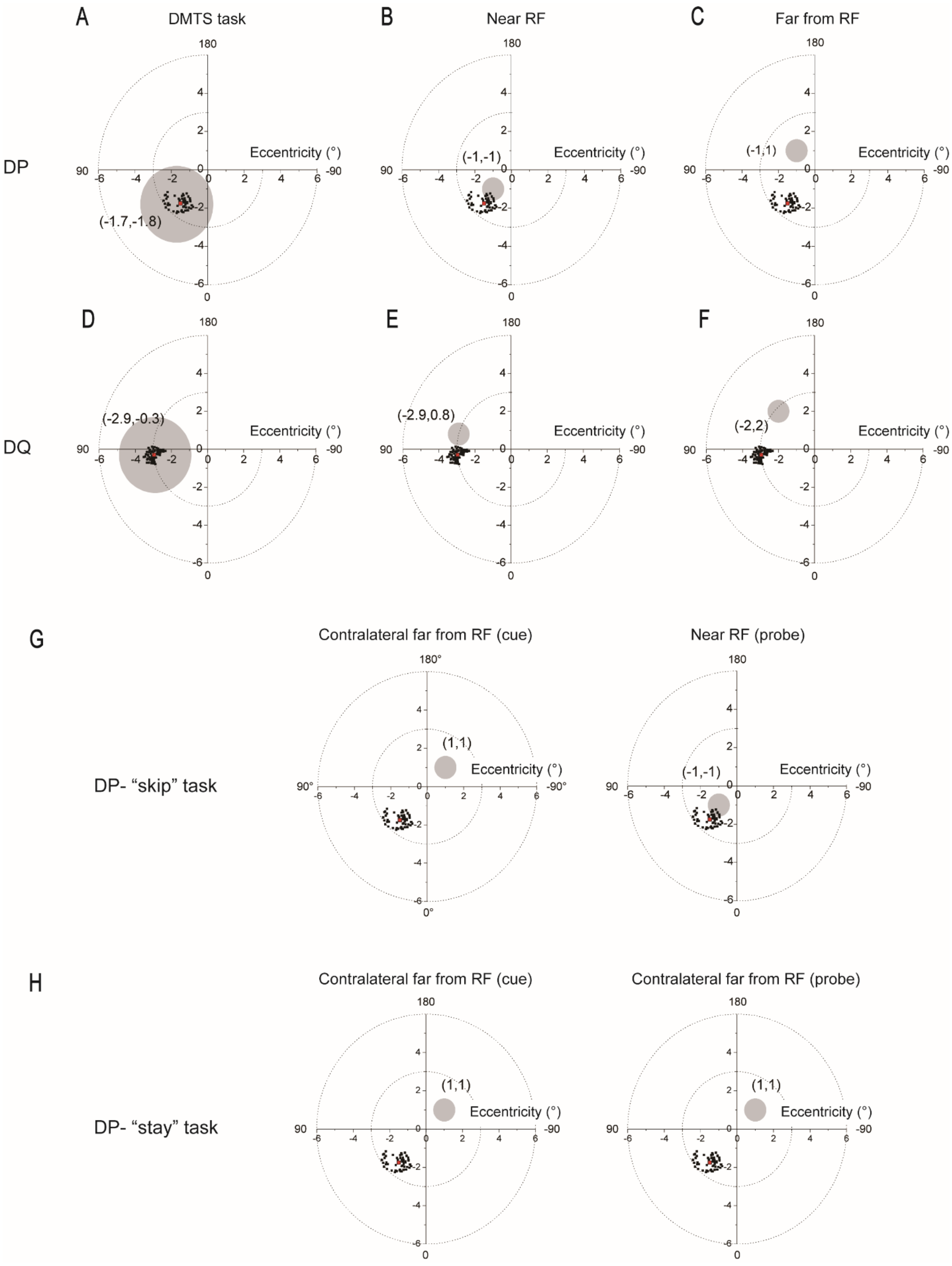
A
DMTS task
B
Near RF
C
Far from RF
DP
(-1.7,-1.8)
(-1,-1)
(-1,1)
D
E
F
DQ
(-2.9,-0.3)
(-2.9,0.8)
(-2,2)
Eccentricity (°)
180
0
90
-90
G
DP- "skip" task
Contralateral far from RF (cue)
Near RF (probe)
(1,1)
(-1,-1)
180°
0°
90°
-90°
H
DP- "stay" task
Contralateral far from RF (cue)
Contralateral far from RF (probe)
(1,1)

fig. S2. Centers of neuronal spatial receptive fields and stimulus locations in DMTS tasks. The black dots represent RF centers, while the red dots indicate the average RF centers of the implanted arrays for monkey DP (A-C) and monkey DQ (D-F). The gray shaded regions display the location and size of the stimuli in each task. The stimulus layouts for the "skip" and "stay" tasks for monkey DP are shown in (G) and (H), respectively.

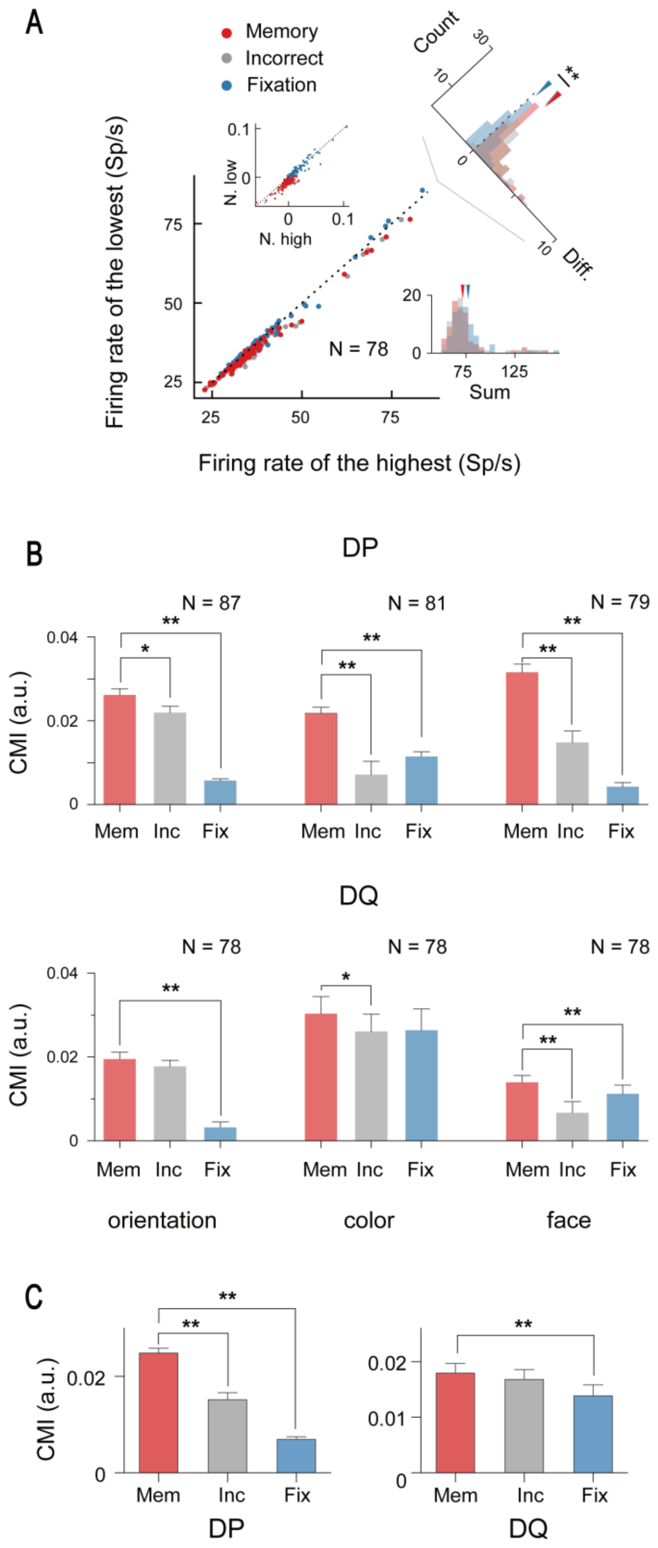

fig. S3. Neuronal responses and CMI values for both monkeys during the delay period. (A) Comparison of averaged highest- firing and lowest-firing to VWM contents in the delay period for the three task conditions (as in Fig. 1E) among all valid electrodes in monkey DQ's orientation DMTS task. **(B)** CMI values for both monkeys were calculated during the delay period across three distinct types of memory stimuli in the DMTS experiments, specifically for orientation, color, and

face picture DMTS tasks. **(C)** CMI values were calculated for both monkeys in three conditions during the delay period (700-1700 ms after cue onset) and combined across all valid electrode sites for orientation, color, and face DMTS tasks. This CMI calculation was performed using a subset of randomly selected trials from correct DMTS tasks to designate the highest-lowest conditions and then applying this designation to the remaining data. The electrode counts were DP: N = 87/81/79 and DQ: N = 78/78/78 for the orientation, color, and face DMTS tasks, respectively. 20-fold cross-validation, iteration =200. If the number of incorrect trials in any experiment was less than 5%, then for that experiment, we conducted n-fold sampling based on the smallest ratio of incorrect to correct trials. "n" specifically denotes the ratio of the number of correct trials to the number of trials in the content condition with the fewest incorrect trials. Within each type of experiment, "Mem" refers to trials where the monkeys answered correctly, "Inc" relates to trials with incorrect responses, and "fix" denotes trials in the fixation task where monkeys received rewards for simply fixating on a point on the screen. All error bars represent ±SEM, with ** $P < 0.01$ and * $P < 0.05$.

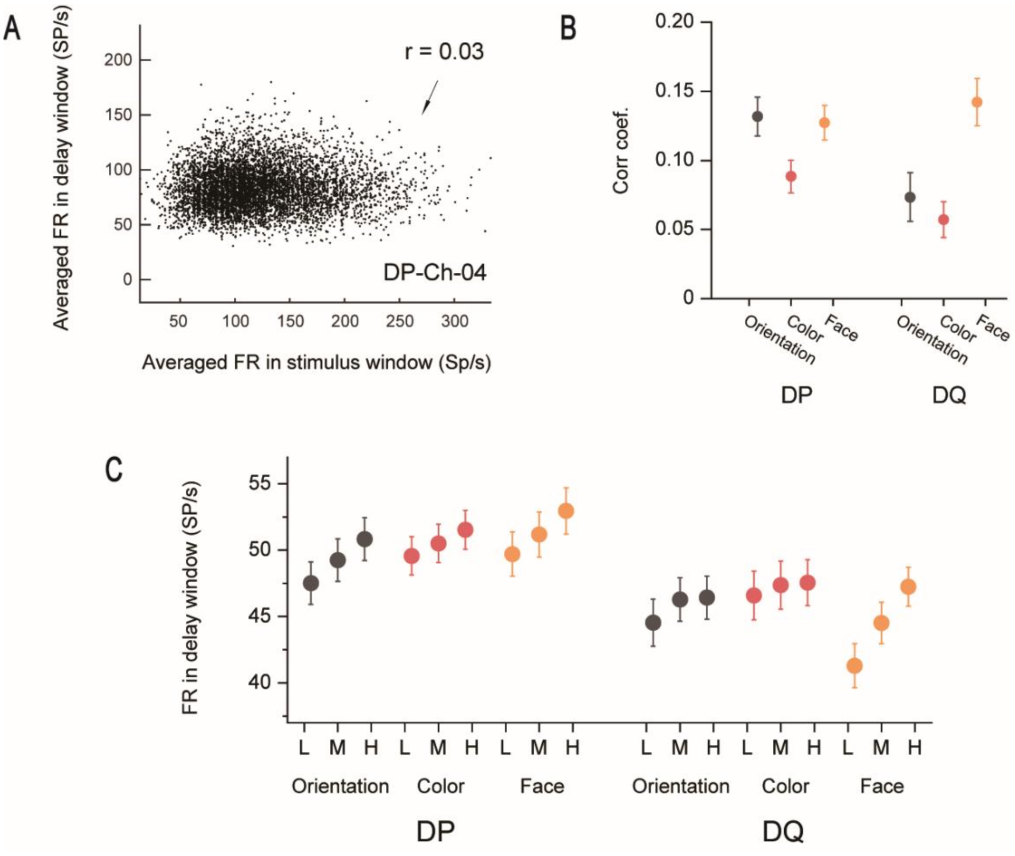

fig. S4. **(A)** Trial-by-trial response at an example electrode. The horizontal axis represents responses within the stimulus period (0-200 ms after cue onset), while the vertical axis represents responses within the delay period (700-1700 ms after cue onset). **(B)** The correlation coefficients between the response during the stimulus period and the delay period across three DMTS experiments of the two monkeys, averaged over all valid electrodes. **(C)** Average responses during the delay period for trials with low, medium, and high responses in the stimulus period. Here, L/M/H represents trials in which the lowest 25%, middle 25%-75%, and highest 25% of the responses occurred in each DMTS experiment. All error bars represent ± SEM.

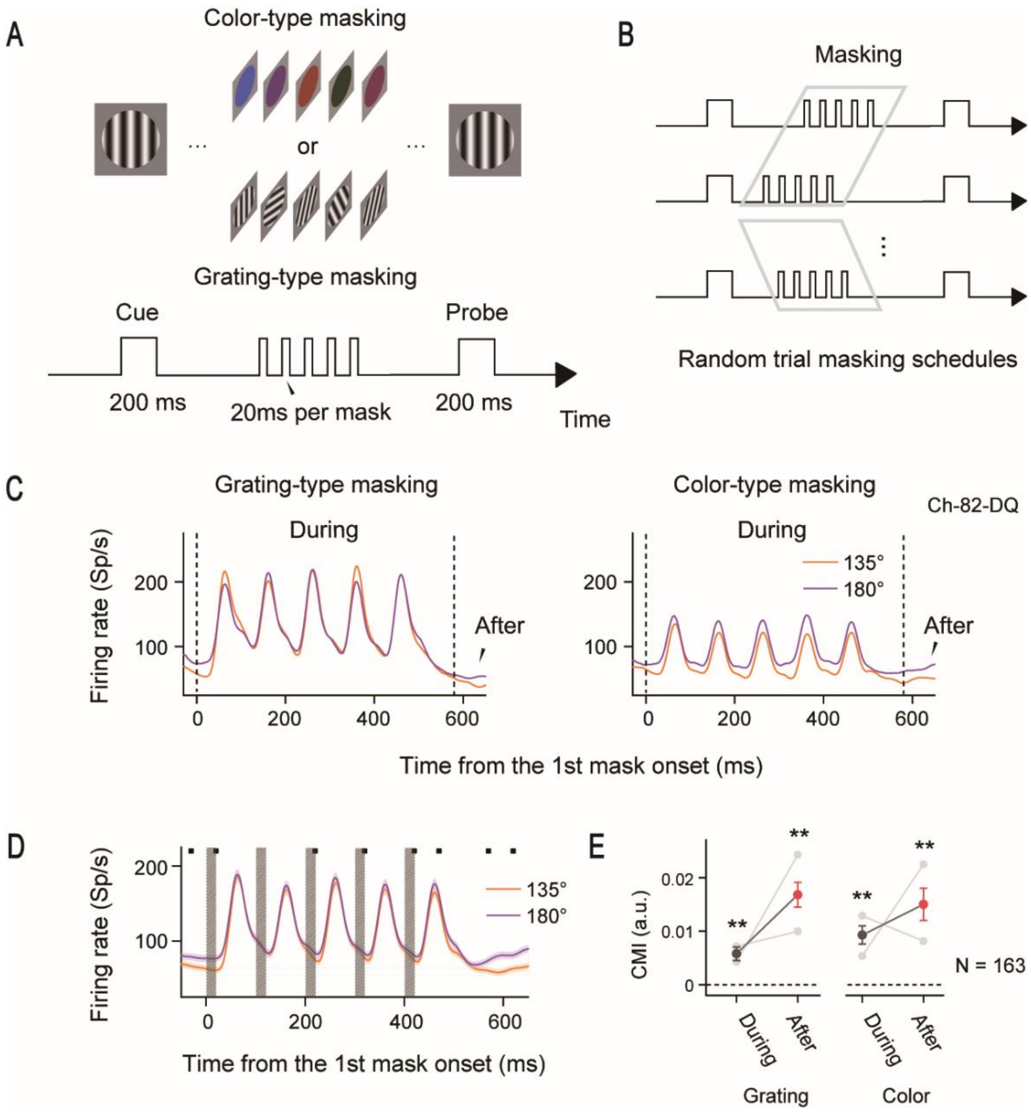

fig. S5. Resilience of VWM content differentiation to interference. (A) In the masking delayed match-to-sample (DMTS) task, the monkey was required to maintain consistent fixation on a central point on the screen. Each trial was initiated by the monkey pulling a lever. Upon this action, an oriented grating, known as the "cue", appeared for 200 ms. Following a 1600 ms interval after the cue's disappearance, another stimulus, termed the "probe", was presented. The monkey's task was to discern via the lever if the cue and probe were the same or different. Notably, during the delay period, five mask stimuli were presented at random time windows, each lasting 20 ms with an intermask interval of 80 ms, totaling 420 ms of masking time. These mask stimuli included gratings (grating-type masking) or uniform color patches (color-type masking), each with a 50-50 probability of appearing during a trial. Specifically, there were eight possible orientations for the gratings and twelve unique colors for the masking patches. The specific orientations or colors for the five mask stimuli within a trial were randomized. **(B)** In different trials, the masking stimulus appeared at varying times. To avoid the influence of off-response from previous cue stimuli on the response profiles of the masking stimulus, we analyzed only trials where the masking stimulus

appeared within the 400–1200 ms window after cue onset. **(C)** (Left) Examples of neuronal responses to grating-type masking data. (Right) Examples of neuronal responses to color-type masking data. **(D)** (Left) First-mask-triggered average activity in monkey DQ at an example electrode site in the masking DMTS task (N = 1862 trials for 135°, N = 1953 trials for 180°; $P < 0.01$ for all marked 50 ms time windows). The gray shaded regions indicate masking windows, and Sp/s represents spikes per second. **(E)** CMI values in grating-type masking data and color-type masking data during and after the masking sequence (during: 0 to 580 ms, after: 580 to 680 ms; N = 163 electrodes, all $P < 0.01$); gray lines (N = 85 for DP and N = 78 for DQ) represent CMI values for each monkey. The error bars denote the SEM, and ** $P < 0.01$.

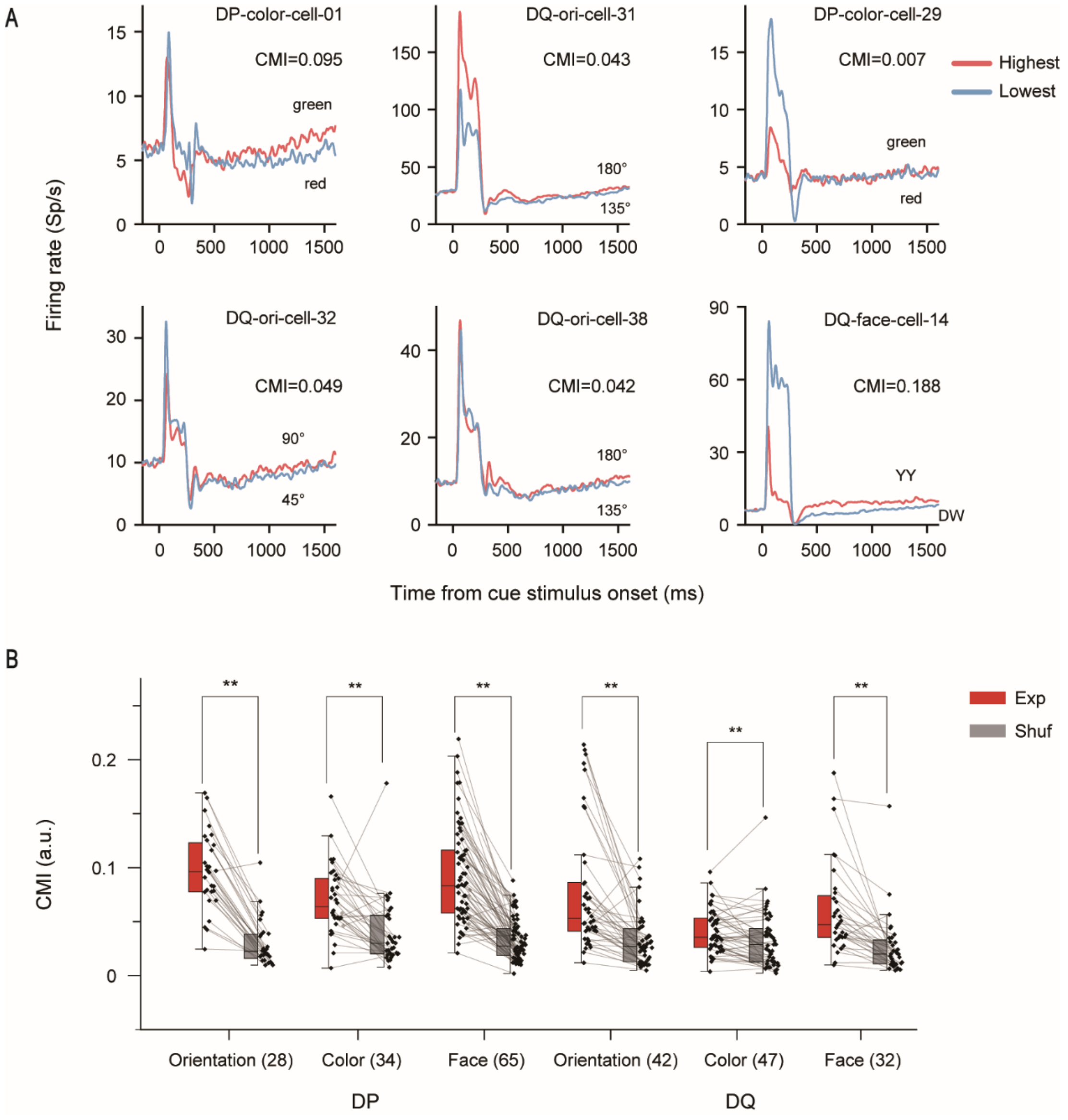

fig. S6. Validation of visual working memory content modulation at the single-unit level in V1. (A) Six examples of single-unit response profiles during the delayed match-to-sample (DMTS) experiment. The red and blue curves represent the conditions with the highest and lowest responses, respectively, during the delay period (700-1700 ms after cue onset), which we defined as the "highest/lowest content." Additionally, the specific memory content corresponding to each condition is annotated alongside the response curves. **(B)** The content modulation index (CMI) values for single units calculated across six DMTS experiments involving two monkeys (red boxes) compared to the chance distribution of CMI values (gray boxes) calculated using the same procedure but with shuffled trials. On the horizontal axis, next to each experiment's name, the number of single units involved in the corresponding experiment is indicated within parentheses. "Exp" refers to experimental data, "Shuf" indicates data with shuffled trials, and "Sp/s" represents spikes per second. ** $P < 0.01$.

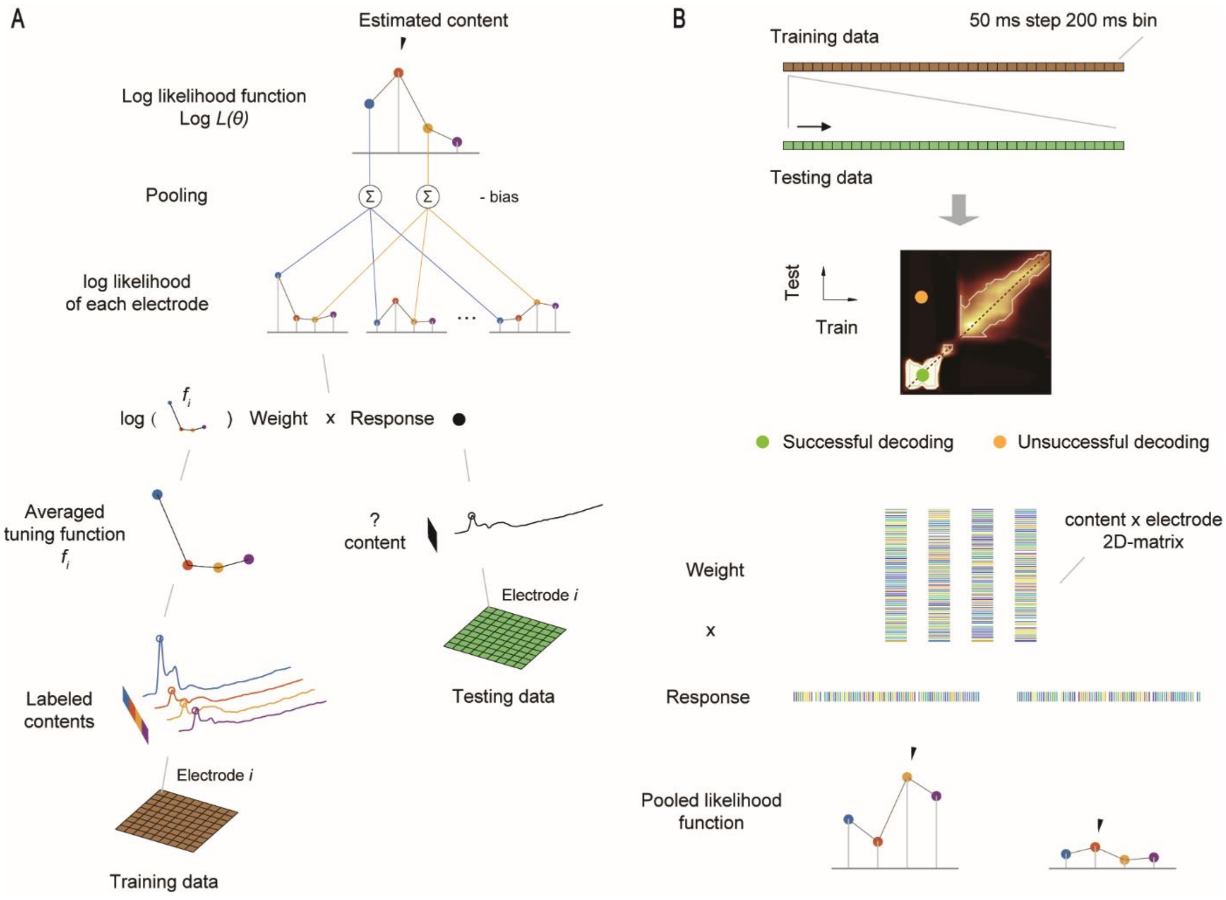

fig. S7. (A) The architecture of the decoding process based on the PID model. We derived a tuning function for each valid electrode from the training data and, after taking logarithms, obtained the weights for each electrode's response. These weights were then multiplied by the response of each electrode in the test data. After pooling these products and subtracting the bias term, which is the average tuning curve of all electrodes from the training data, we obtained the log likelihood function. The label corresponding to the highest value of this function is the estimated content label. **(B)** Schematic of cross-temporal decoding (top) and diagram illustrating the computational process yielding successful/unsuccessful decoding outcomes (bottom). From each time window, we extracted the average neuronal response to train the weights. These weights were subsequently used to estimate the VWM content labels for all the time windows. In the lower part of the graph, we illustrated the potential successful or unsuccessful decoding outcomes when using data from the same time window for training but different time windows for testing. This variation in outcomes is attributed to neurons displaying different firing intensities and patterns across various time windows. Here, weights and neuronal responses were normalized and trimmed to provide a simplified schematic representation.

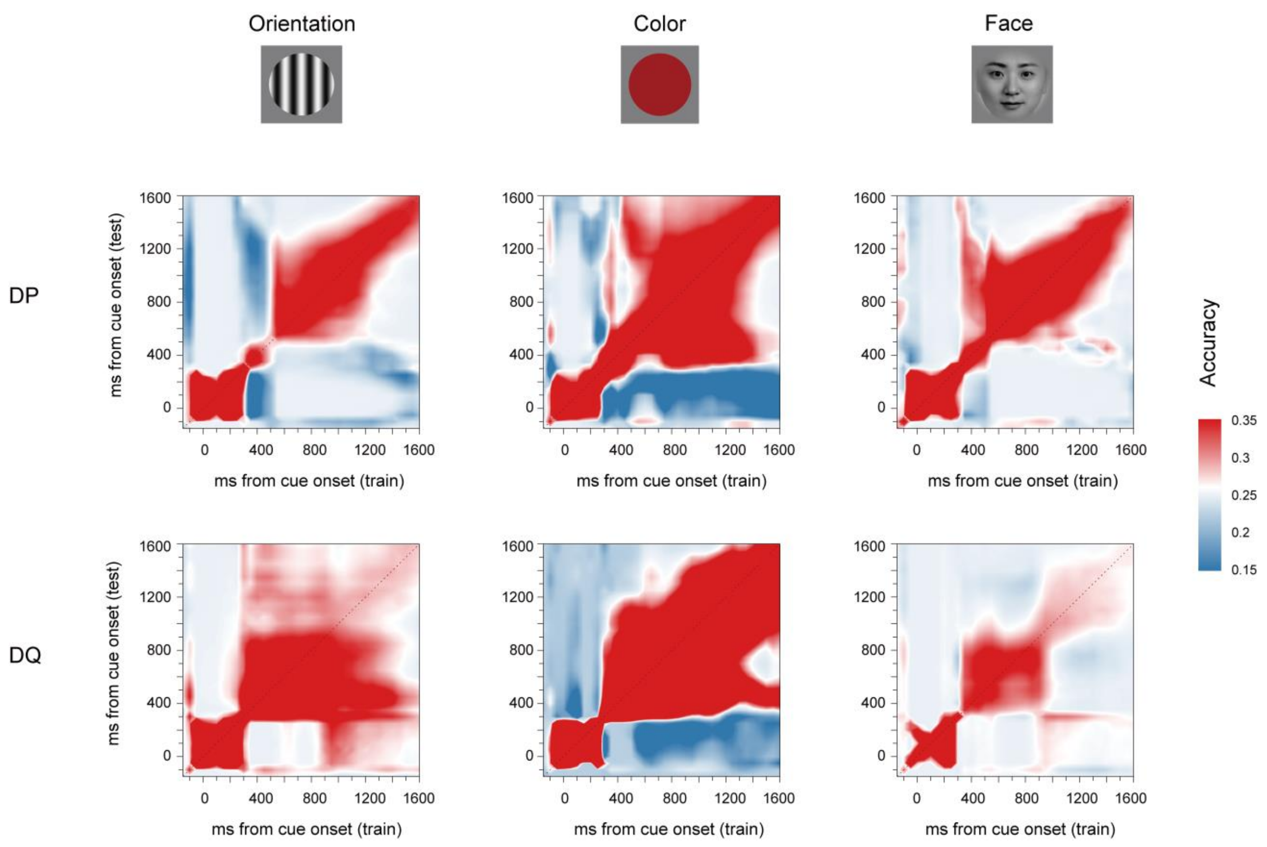

fig. S8. Cross-temporal decoding results for VWM content. Separate classifiers were constructed using neuronal population activity in each temporal sliding window (200 ms wide and 50 ms steps), revealing information coding dynamics. The results for the orientation, color, and face DMTS tasks are displayed in the three rows. The color bar is consistent across all six subfigures.

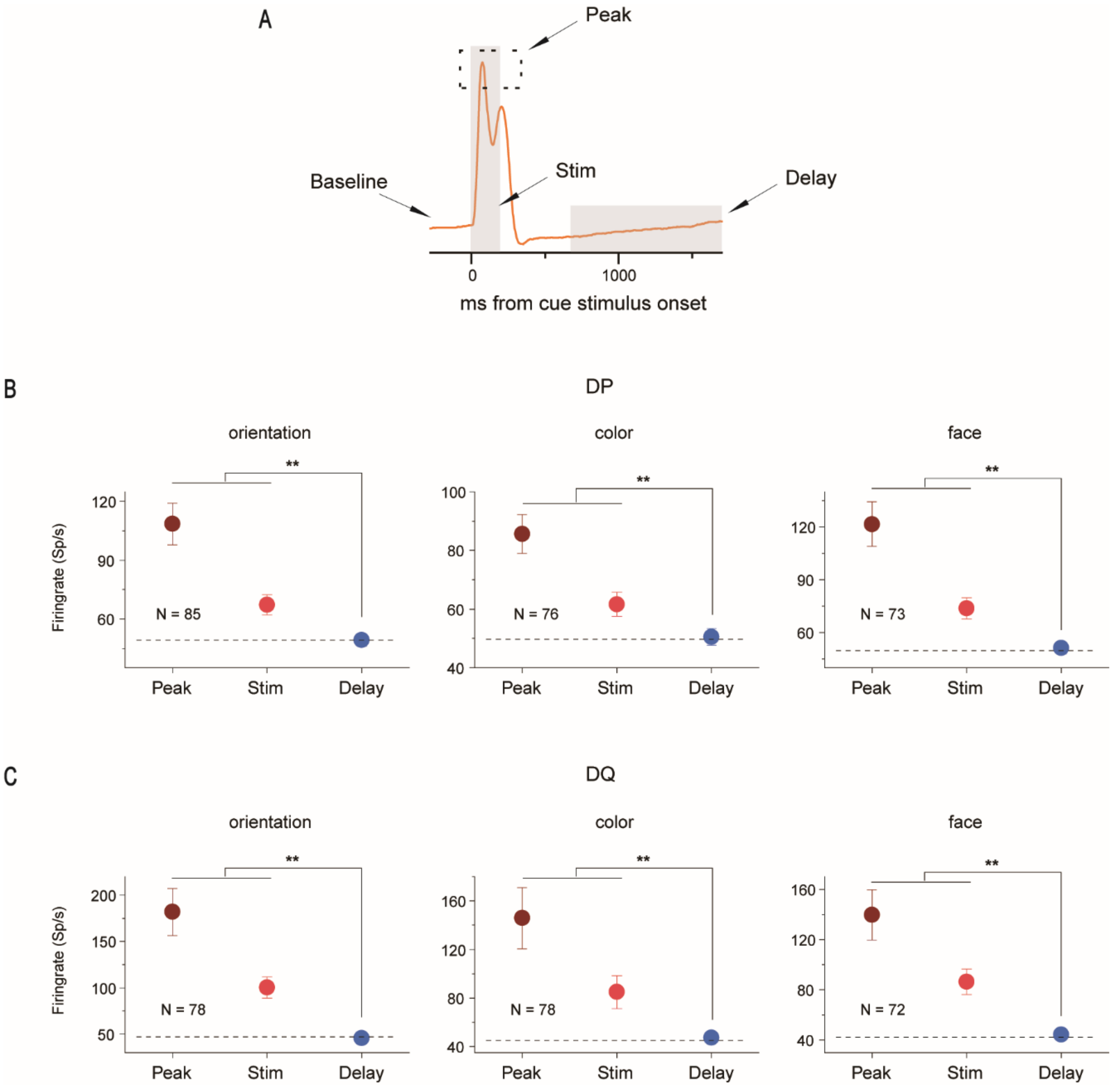

fig. S9. Neuronal firing rates during stimulus and delay periods in three memory tasks for both monkeys. (A) Illustration of the selected time windows for electrode responses. Here, "baseline" (the gray dashed lines) refers to the average response during the 150-0 ms before the cue stimulus, "peak" refers to the maximum response within 0-200 ms after cue onset, "stim" refers to the average response within 0-200 ms after stimulus onset, and "delay" refers to the average response during the 700-1700 ms after stimulus onset. **(B)** The response of monkey DP at the "peak", "stim", and "delay" time windows or points, with dashed lines indicating the average baseline response. **(C)** The same as (B) but showing the data for monkey DQ. Sp/s stands for spikes per second. The error bars denote the SEM, and ** $P < 0.01$.

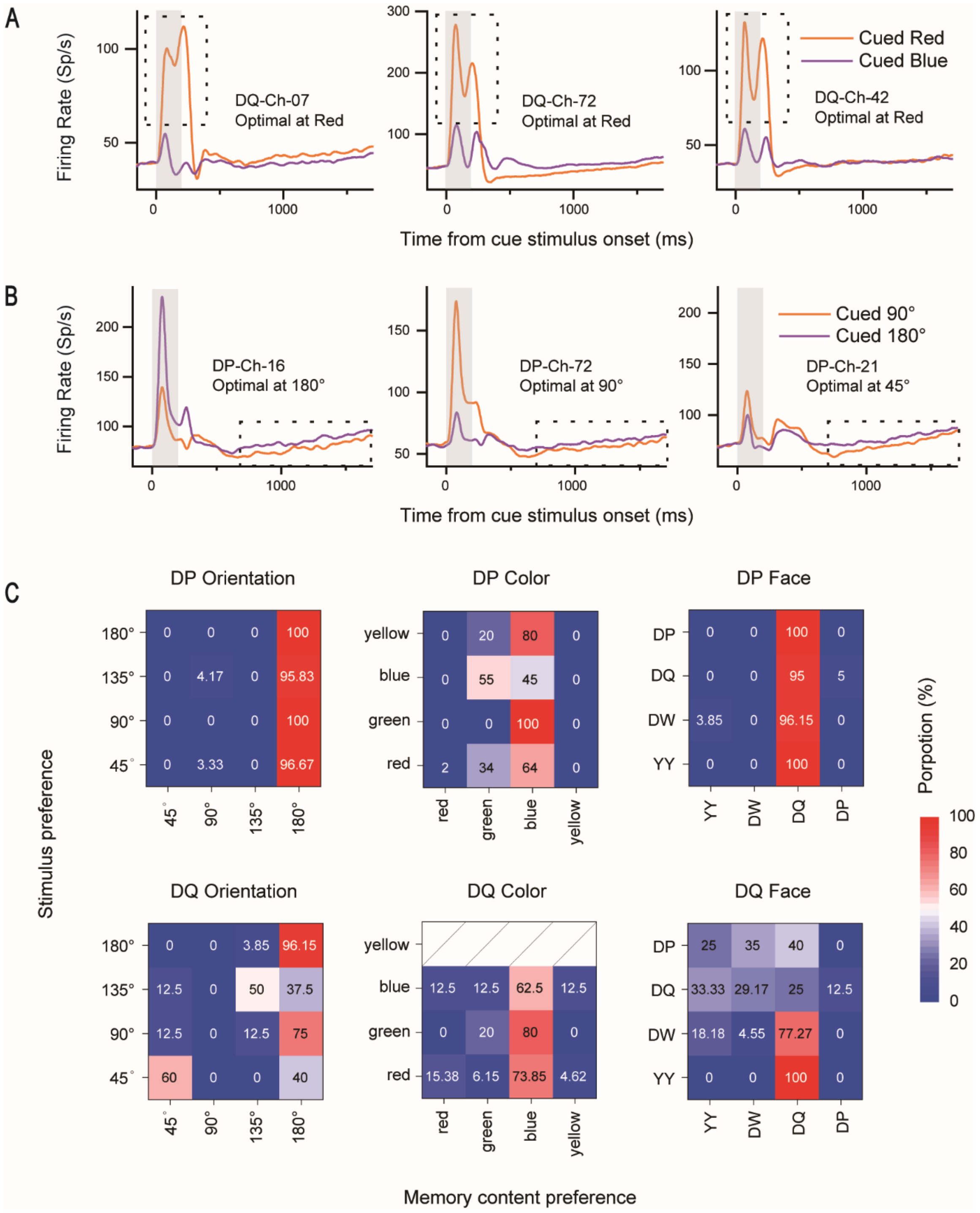

fig. S10. Example neuronal activity demonstrating transformative information coding during the VWM task. (A) Neuronal activity from example electrodes displaying the same visual stimulus preference but without maintaining a similar differential pattern during the delay period. Sp/s stands for spikes per second. **(B)** Neuronal activity from example electrodes preserving consistent content representation rankings during the delay period, regardless of their differing visual stimulus preferences. **(C)** Preference descriptions of valid sites across all 6 memory experiments. Each row incorporates data from sites with the highest firing rates under the stimulus conditions labeled on the vertical axis during the stimulus period. The colors within the squares

represent the probability of these sites having the highest firing rates under the stimulus conditions labeled on the horizontal axis during the delay period. Each row includes neurons with a preference for a particular stimulus; for example, in the figure displaying results from the color DMTS task, the second row includes neurons that respond most strongly to blue stimuli during the stimulus period (0-200 ms after cue onset). We calculated the percentage of these neurons that responded most strongly to a particular condition during the delay period. Boxes crossed out with a diagonal line indicate the absence of electrodes exhibiting a preference for the corresponding stimulus, such as a preference for a yellow stimulus.

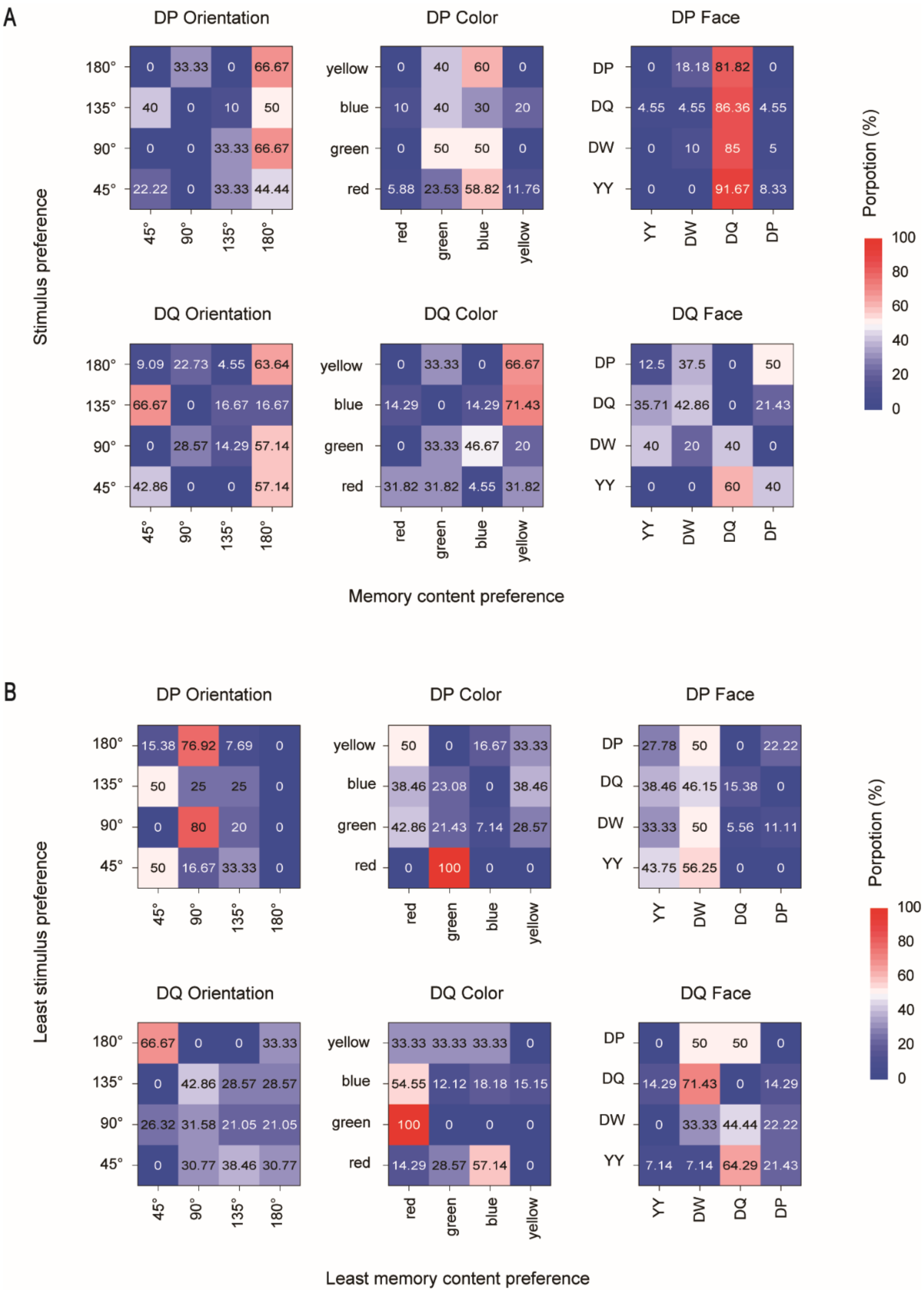

fig. S11. Preference descriptions of single units across all six memory experiments. (A) Similar to fig. S10C but focusing on single units. **(B)** Identical to (A) but for the least preferred stimuli during both the stimulus period and the delay period.

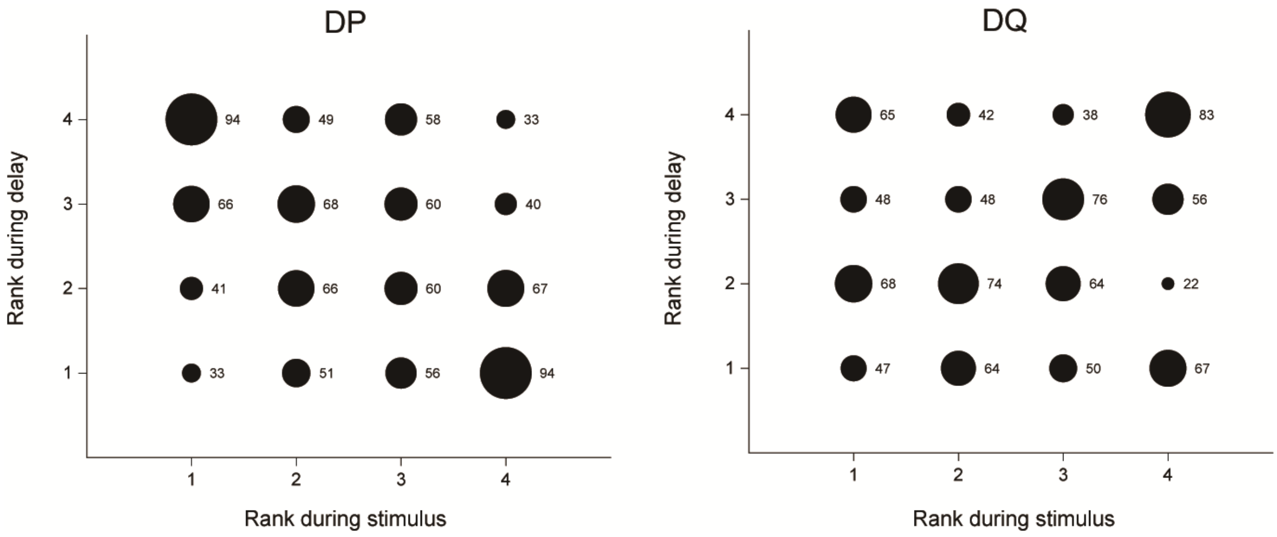

fig. S12. Scatter plot illustrating Spearman's rank correlations of the relationship between stimulus tuning and VWM content tuning. The size of the dots and the corresponding numbers on the right signify the number of electrodes. For instance, if an electrode exhibited the highest response during the stimulus period for the blue content condition across all conditions but was the second lowest for the blue condition during the delay period, it was positioned at the coordinates (4, 2) on the plot. The correlation coefficients are detailed in Table S1. The data from both monkeys did not show any significantly strong positive or negative correlations.

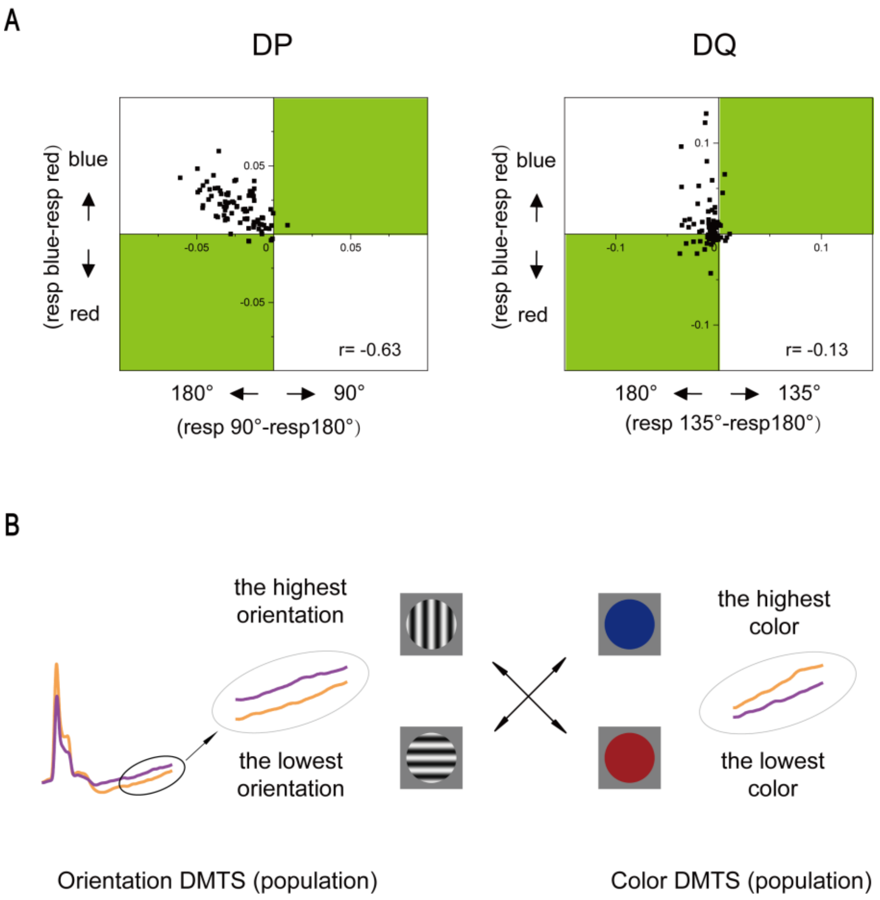

fig. S13 (A) The response preference of neurons during the delay period in the orientation DMTS and color DMTS tasks. In designing the stimulus pairing for the association task, we used antagonistic stimulus pairs, as shown in (B). For instance, the left graph indicates that monkey DP's neurons had greater responses to 180° orientation and blue stimuli during the delay period. Hence, we matched the higher response orientation condition (180°) with the lower response color (red) as a rule (indicated by the grass-green area in the graph). Similarly, we matched the lower response orientation condition (90°) with the higher response color (blue) as a rule. Since there are only two conditions in the association task, common preferences during the DMTS task (such as a preference for both 180° and blue) also led to below-baseline decoding accuracy for color content in the early stages of antagonistic stimulus pairing. **(B)** Conceptual diagram of the rule for designing the antagonistic stimulus pairs in the association task.

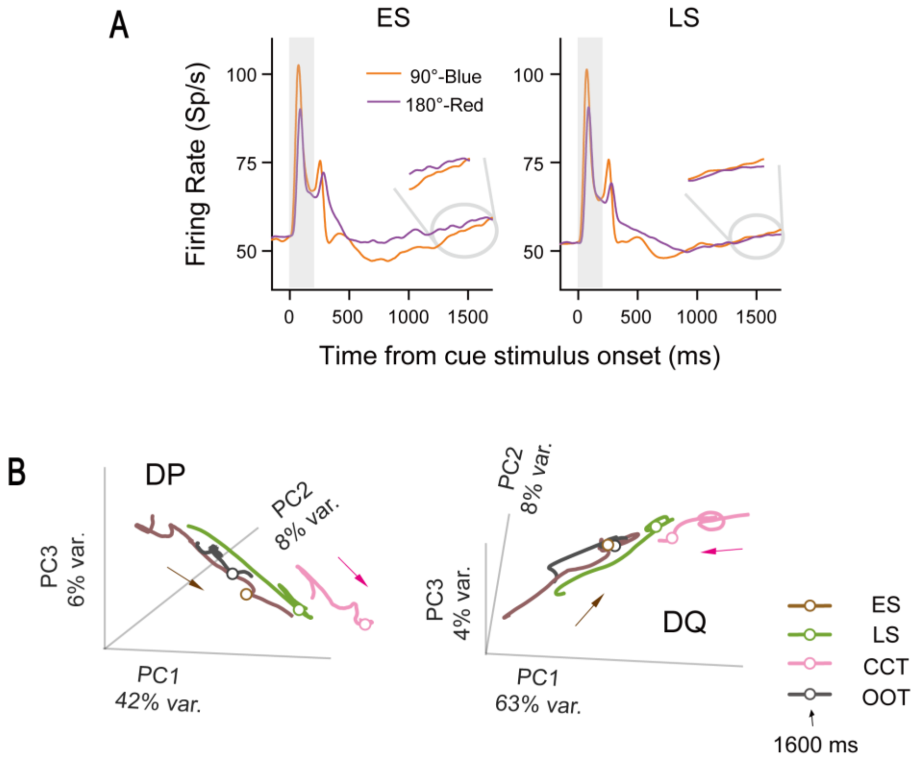

fig. S14. (A) Responses averaged across all electrodes in the early and late stages of the association task, respectively, with an emphasis on the later delay period response. This result indicates that content association impacted neuronal memory maintenance activity but not sensory evoked activity. **(B)** Projections of CMI values onto the first three PCs calculated from the color-color DMTS task. The projections during the 700-1700 ms period following cue stimulus onset are similar to those displayed in Fig. 3G. The proportion of variance explained is indicated along each axis. Arrows represent the direction of time within a trial. The hollow points correspond to the data at 1600 ms after cue stimulus onset. "ES" and "LS" refer to data from trials in the early and late stages of the association task.

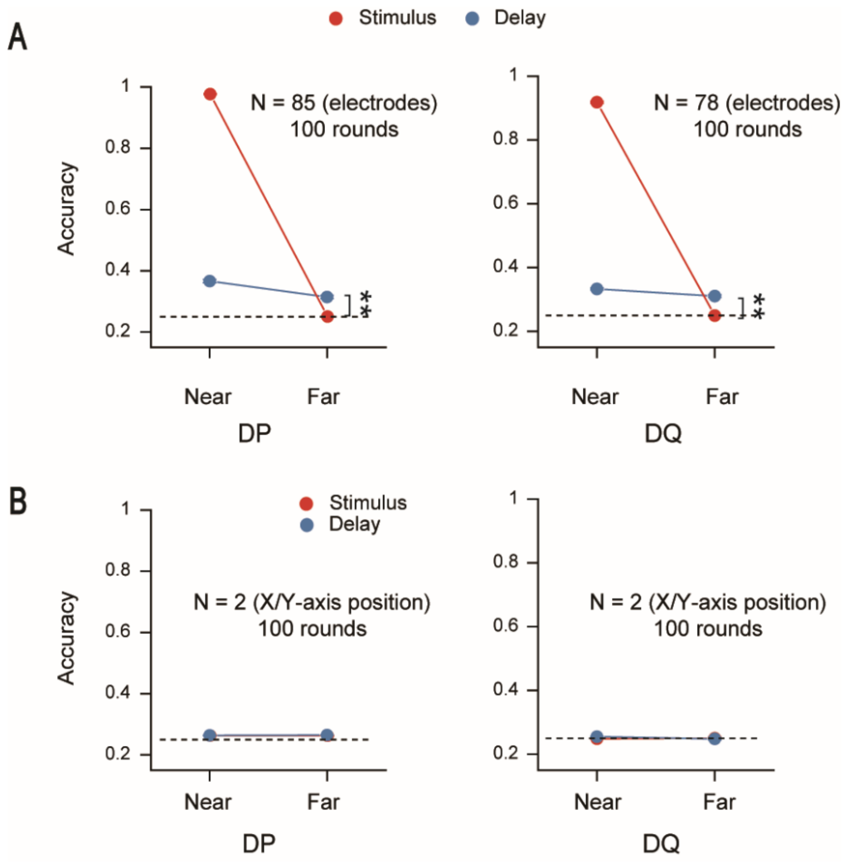

fig. S15 Validation of the spatial extent of visual working memory representation in V1 exceeding that of visual stimulus representation using a linear decoder. (A) Results of decoding working memory content using a linear SVM decoder trained on MUA signals from all valid electrodes in the "near RF" task applied to both the "near RF" and "far from RF" tasks. **(B)** Results of decoding working memory content using a linear SVM decoder trained on eye movement signals from the "near RF" task, with "N = 2" representing the two dimensions of eye movement, specifically the x- and y-axis positions. We randomly selected 1200 trials from the "near RF" task experiments for training (300 per content condition). The trained decoder was then applied to the randomly selected 1200 trials in the remaining trials of the "near RF" and "far from RF" tasks. This process of random iteration was repeated 100 times. In (B), the overlapping blue and red dots and lines may cause parts of the red lines to be obscured. This decoding analysis was conducted separately for both the stimulus period and the delay period. ** $P < 0.01$, and the dashed line represents the chance level.

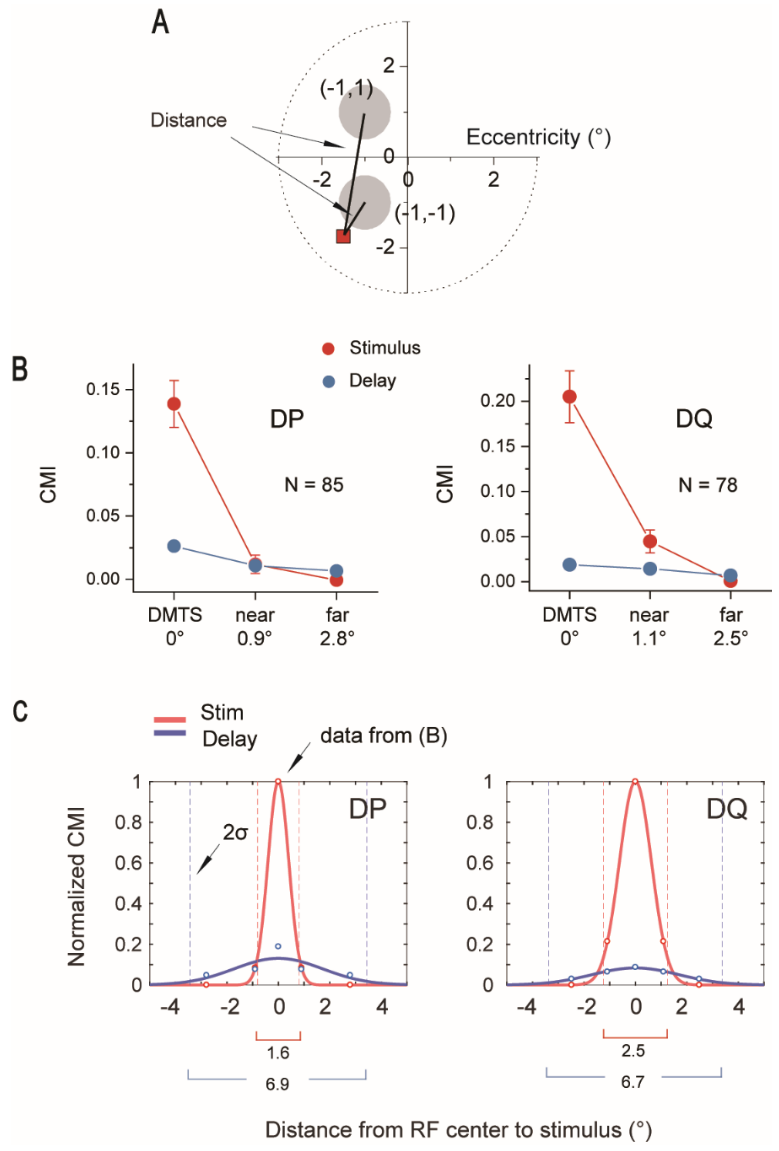

fig. S16 Estimation of the spatial extent of VWM content modulation. (A) Illustration showing the distance between the center of the visual stimulus and the center of the neuron's receptive field. **(B)** CMI values were calculated using data from both monkeys, with the inclusion of additional data from the orientation DMTS task. This task also provided the basis for designating the highest and lowest conditions in CMI calculations, differing from the designation used in Fig. 4, to estimate the modulation of visual stimuli and memory content at the center of the receptive field. The horizontal axis additionally marks the distance of the visual stimulus from the electrode's receptive field for each experiment. **(C)** Gaussian curve fitting applied to the data points from (B). The Gaussian function constrains CMI values to be greater than 0. The data points are symmetrically processed around x=0 to form a complete Gaussian shape. The 4σ of the fitted Gaussian curve is denoted as the spatial extent (diameter) of the visual stimulus and memory content representation, labeled below the horizontal axis.

Animal name	Orientation	Color	Face	Pooled
DP	-0.37 (85)	-0.41 (76)	0.01 (73)	-0.26 (234)
DQ	0.51 (78)	-0.19 (78)	-0.19 (72)	0.04 (228)

Table S1. Mean Spearman's rank correlation coefficients of the relationship between stimulus tuning and VWM content tuning for individual animals (DP and DQ). The term "pooled" refers to the average correlation coefficient calculated by combining the results from the three experiments. The numbers in parentheses represent the number of valid electrodes analyzed. These results do not indicate any stable similarity in encoding patterns across the two coding phases.
